# Gene expression profile indicates involvement of NO in *Camellia sinensis* pollen tube growth at low temperature

**DOI:** 10.1186/s12864-016-3158-4

**Published:** 2016-10-18

**Authors:** Junting Pan, Weidong Wang, Dongqin Li, Zaifa Shu, Xiaoli Ye, Pinpin Chang, Yuhua Wang

**Affiliations:** College of Horticulture, Nanjing Agricultural University, Nanjing, 210095 China

**Keywords:** Pollen tube, Low temperature, Nitric oxide, *Camellia sinensis*, RNA-Seq, Transcriptome

## Abstract

**Background:**

Nitric oxide (NO) functions as a critical signaling molecule in the low-temperature stress responses in plants, including polarized pollen tube growth in *Camellia sinensis*. Despite this, the potential mechanisms underlying the participation of NO in pollen tube responses to low temperature remain unclear. Here, we investigate alterations to gene expression in *C. sinensis* pollen tubes exposed to low-temperature stress and NO using RNA-Seq technology, in order to find the potential candidate genes related to the regulation of pollen tube elongation by NO under low-temperature stress.

**Results:**

Three libraries were generated from *C. sinensis* cv. ‘Longjingchangye’ pollen tubes cultured at 25 °C (CsPT-CK) and 4 °C (CsPT-LT) or with 25 μM DEA NONOate (CsPT-NO). The number of unigenes found for the three biological replications were 39,726, 40,440 and 41,626 for CsPT-CK; 36,993, 39,070 and 39,439 for CsPT-LT; and 39,514, 38,298 and 39,061 for CsPT-NO. A total of 36,097 unique assembled and annotated sequences from *C. sinensis* pollen tube reads were found in a BLAST search of the following databases: NCBI non-redundant nucleotide, Swiss-prot protein, Kyoto Encyclopedia of Genes and Genomes, Cluster of Orthologous Groups of proteins, and Gene Ontology. The absolute values of log_2_Ratio > 1 and probability > 0.7 were used as the thresholds for significantly differential gene expression, and 766, 497 and 929 differentially expressed genes (DEGs) were found from the comparison analyses of the CK-VS-LT, CK-VS-NO and LT-VS-NO libraries, respectively. Genes related to metabolism and signaling pathways of plant hormones, transcription factors (TFs), vesicle polarized trafficking, cell wall biosynthesis, the ubiquitination machinery of the ubiquitin system and species-specific secondary metabolite pathways were mainly observed in the CK-VS-LT and CK-VS-NO libraries.

**Conclusion:**

Differentially expressed unigenes related to the inhibition of *C. sinensis* pollen tube growth under low temperature and NO are identified in this study. The transcriptomic gene expression profiles present a valuable genomic tool to improve studying the molecular mechanisms underlying low-temperature tolerance in pollen tube.

**Electronic supplementary material:**

The online version of this article (doi:10.1186/s12864-016-3158-4) contains supplementary material, which is available to authorized users.

## Background

Tea (*Camellia sinensis* (L.) O. Kuntze) is a popular evergreen beverage tree, grown worldwide in different agro-climatic between 45°N and 34°S latitude [1]. Low-temperature stress is one of the most critical abiotic stresses that negatively influence the growth, development and geographical distribution of tea plants [2]. Previous studies have noted that low temperature lead to a weakened photosynthetic capacity and strengthened respiration rates [3], and a total of 105 metabolic processes were changed in tea plants, such as carbohydrate metabolism and amino acid metabolism [4], which ultimately decreased the yield and quality of the tea plants. Thus, finding the mechanisms of improving the tolerance of *C. sinensis* to low temperature is of great importance. Recently, certain molecular mechanisms driving the responses of tea plants to low temperature, such as Ca^2+^ signaling, APETALA2/ethylene responsive factor (AP2/ERF), basic helix-loop-helix (bHLH), WRKY and MYB domain-containing protein (MYB) transcription factors (TFs) and zinc finger protein (ZFP), have been demonstrated, particularly in vegetative tissues [5]. These findings provided a solid foundation for further exploring the cold-related genes and improved the understanding of plant tolerance to low-temperature stress. However, previous reports revealed that gene expression in reproductive tissues was different from that in vegetative tissues during cold tolerance [6], implying that reproductive tissue responds to low-temperature stress by different mechanisms [7]. Furthermore, the pollen tube, as a key component of genetic breeding, plays a crucial role in the higher plants’ reproduction, and its growth is highly susceptible to low temperature. For example, certain reports indicate that low temperature clearly reduce pollen tube elongation and disrupt its morphology [8, 9]. Interestingly, Camellia pollen can germinate *in vitro* even at 3 °C, whereas lily pollen cannot germinate at low temperature [10], implying higher tolerance to low temperature in Camellia pollen. Our previous report also demonstrated that *C. sinensis* pollen could germinate and grow at low temperature, although the germination and elongation were both retarded by cold stress [11]. Thus, the basic molecular mechanisms regulating pollen germination and tube elongation in response to low-temperature stress in *C. sinensis* deserve further attention.

Recently, nitric oxide (NO) has gained recognition for its significance in various essential physiological progresses in plants [12], and much attention has been focused on its roles in the plant tolerance to diverse abiotic stresses, including high salt [13], drought [14], heat [15], heavy metal [16], ultraviolet radiation [17], and cold stress [18]. Evidence suggests that NO production regulates diverse responses during cold stress. For example, NO production was increased in *Triticum aestivum* roots under treatment with cold stress [19], whereas it was reduced in *Capsicum annuum* leaves under treatment with low temperature [20]. Furthermore, NO has been confirmed to participate in the anabolic pathway and signal transduction of plant hormones under low-temperature stress. Guo et al. [21] revealed that NO participates in mediating the cold-induced expression of an enzyme related to the ethylene precursor *MfSAMS1* (S-adenosylmethionine synthetase) by interacting with abscisic acid (ABA) and H_2_O_2_ in *Medicago sativa* subsp. *Falcate*. Recent reports have shown that NADPH oxidase inhibitors and reactive oxygen species scavengers reduced 24-epibrassinolide (EBR)-induced NO production, whereas EBR-induced H_2_O_2_ synthesis was not sensitive to NO. Decreased NO content inhibited EBR-induced low-temperature tolerance and partly inhibited EBR-induced expression of genes coding for several antioxidant enzymes and their relative activities in cucumber, suggesting that NO plays key roles in the H_2_O_2_-dependent induction of plant tolerance to low-temperature stress by brassinosteroids (BRs) [22]. Additionally, accumulating evidence has suggested that TFs are modulated by NO during cold stress. For instance, the C repeat binding factor 1 (CBF1) and CBF3 TFs are accumulated because of NO during *Arabidopsis* responding to low temperature [23]. Similarly, application of the NO donor sodium nitroprusside (SNP) increased *LeCBF1* expression, whereas nitro-arginine (a NO synthesis competitive inhibitor) reduced its expression in tomato fruits during cold tolerance [24]. A recent study has indicated that NO participates in ABA-dependent cold signaling by negatively regulating the DNA binding of MYB2 by S-nitrosylation [25]. However, NO’s participation in low-temperature response is controlled by sophisticated signaling crosstalk for which the potential molecular mechanisms are still undeciphered.

As shown in the previous reports, NO plays crucial roles in pollen tube tip growing, for example its participation in the pollen tube growth regulation and reorientation in lily and *Arabidopsis* [26, 27]. In addition, extracellular nucleotides suppress *Arabidopsis* pollen germination and tube growth, which is modulated partly through the NO signaling pathway [28]. Wang et al. [29] reported that NO affects the cell wall construction in pollen tubes through the alteration of extracellular Ca^2+^ influx and F-actin organization, which consequently affects pollen tube elongation in *Pinus bungeana*. More interestingly, our previous report revealed that NO production from a NO synthase (NOS)-like enzymatic reaction enhanced the inhibiting effect of low-temperature stress on pollen germination and tube elongation in *C. sinensis*, and decreased free proline accumulation, which was accomplished partly through the guanosine 3’, 5’-cyclic monophosphate (cGMP) signaling pathway [11]. However, the downstream signaling and function mechanisms behind the NO-induced reduction of pollen tube elongation under low-temperature stress in *C. sinensis* remain unclear.

High-throughput transcriptomic analysis techniques can provide an overview of expression changes in response to biotic and abiotic stresses. In particular, RNA-Seq has become a widely used method for the analysis of species whose genome sequence is not yet available [30]. To understand the potential mechanisms driving NO-modulated pollen tube growth during exposure to low-temperature stress, we utilized the Illumina HiSeq™2000 platform to sequence the transcriptomes of *C. sinensis* pollen tubes cultured at 25 °C and low temperature and exposed to exogenous NO treatment, and all of the experiments were replicated three times to guarantee the reliability of the results in this study. Furthermore, the differential gene expression from the transcriptomes of the three treatments was systematically examined, the differentially expressed genes (DEGs) related to plant hormone signaling pathways, TFs, vesicle polarized trafficking, cell wall biosynthesis, the ubiquitination machinery of the ubiquitin system and species-specific secondary metabolite pathways were identified and analyzed. The assembled, annotated transcriptome will present a useful genomic resource for facilitating the investigations on the molecular mechanisms of NO’s involvement in pollen tube tolerance to low-temperature stress.

## Results

### Illumina sequencing and *de novo* assembly

**Fig. 1:**
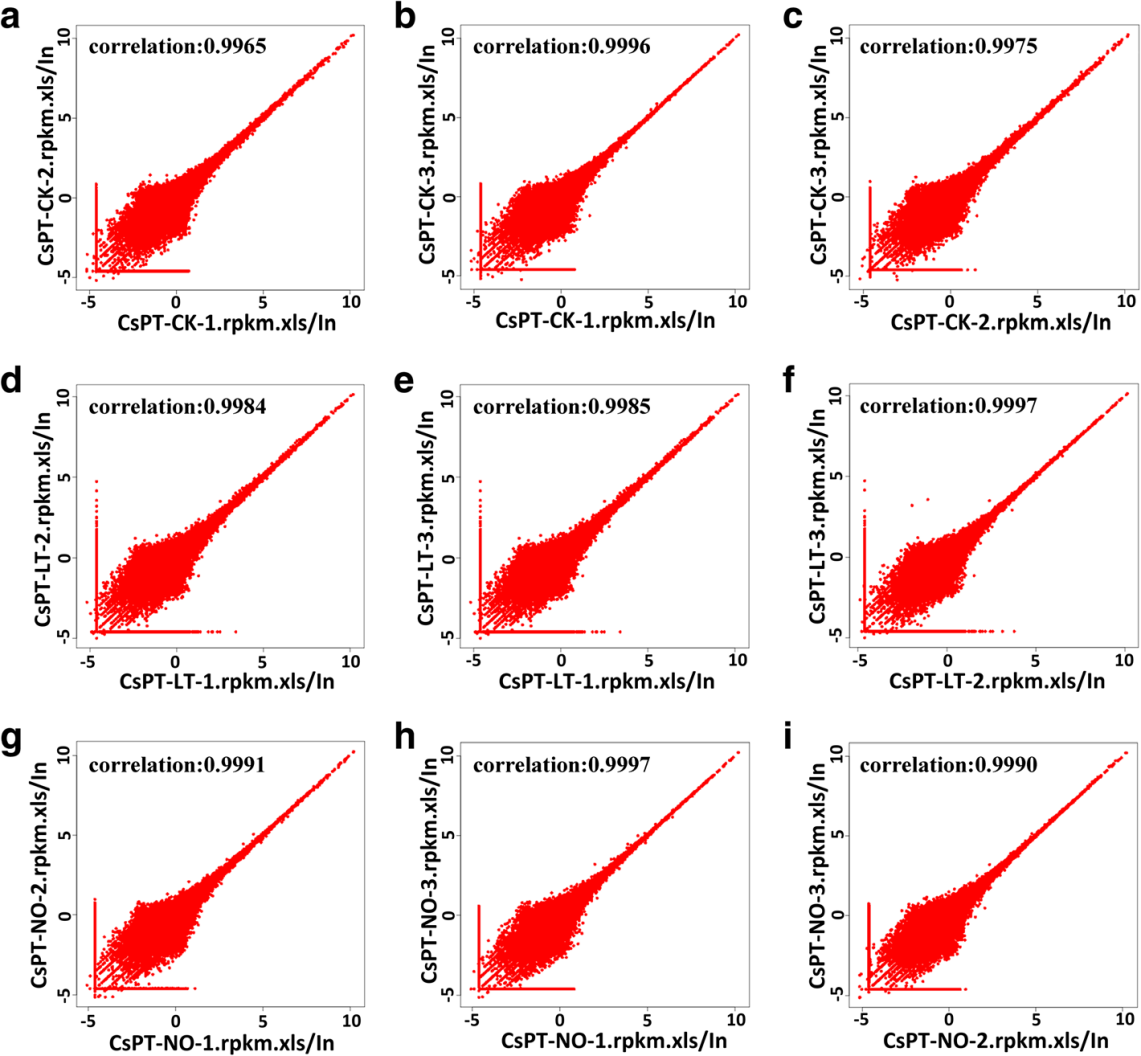
Pearson correlation analysis of the replicates from the control (CK), low-temperature and NO treatments on the *Camellia sinensis* pollen tubes. **a**, **b** and **c** are analyses of the CK treatment; **d**, **e** and **f** are analyses of the low-temperature (LT) treatment, and **g**, **h** and **i** are analyses of the NO donor DEA NONOate (NO) treatment.

To study the transcriptome expression of pollen tubes, *C. sinensis* pollen tubes were cultured under the following conditions: 25 °C (CK), 4 °C (LT) and 25 μM DEA NONOate (NO). Three libraries (CsPT-CK, CsPT-LT, and CsPT-NO) were designed for RNA-Seq using an Illumina HiSeq^TM^2000 genome analyzer, and all of the experiments were replicated three times. After cleaning and performing quality checks, 55.5, 52.4, and 51.3 million clean reads were obtained for CsPT-CK1, CsPT-CK2 and CsPT-CK3 (Accession number SRR3270364, SRR3270376 and SRR3270829 for library CsPT-CK1, CsPT-CK2 and CsPT-CK3), respectively; 50.2, 51.8, and 51.4 million clean reads were obtained for CsPT-LT1, CsPT-LT2 and CsPT-LT3 (Accession number SRR3270928, SRR3270974 and SRR3270993 for library CsPT-LT1, CsPT-LT2 and CsPT-LT3), respectively; and 51.9, 54.5, and 53.7 million clean reads were obtained for CsPT-NO1, CsPT-NO2 and CsPT-NO3 (Accession number SRR3270997, SRR3271001 and SRR3271002 for library CsPT-NO1, CsPT-NO2 and CsPT-NO3), respectively. The Q20 percentage was > 97.77 % in each library as shown in Table 1. The clean reads were assembled into 60,611, 61,246 and 63,117 contigs with mean lengths of 333, 335 and 331 nt for CsPT-CK, respectively; 56,102, 58,622 and 60,547 contigs with mean lengths of 343, 340 and 333 nt for CsPT-LT, respectively; and 59,750, 58,884 and 59,550 contigs with mean lengths of 331, 335 and 333 nt for CsPT-NO, respectively. These contigs were further assembled into 39,726, 40,440 and 41,626 unigenes with mean lengths of 648, 650 and 660 nt for CsPT-CK, respectively; 36,993, 39,070 and 39,439 unigenes with mean lengths of 650, 664 and 657 nt for CsPT-LT, respectively; and 39,514, 38,298 and 39,061 unigenes with mean lengths of 647, 650 and 639 nt for CsPT-NO, respectively (Table 2). These unigenes size distribution was displayed in Additional file 1: Figure S1. As shown in Fig. 1, the Pearson product moment coefficient was > 99.65 % in each library. All above data indicated that the quality of throughput and sequencing was high enough for further analysis.

**Table 1:** Summary of the output in the transcriptomes of pollen tubes

Sample	Total raw reads	Total clean reads	Total clean nucleotides (nt)	Q20 percentage	N percentage	GC percentage
CsPT-CK-1	54,971,748	51,933,988	4,674,058,920	97.92 %	0.00 %	45.44 %
CsPT-CK-2	57,928,046	54,556,816	4,910,113,440	97.92 %	0.00 %	45.23 %
CsPT-CK-3	56,727,452	53,686,756	4,831,808,040	97.93 %	0.00 %	45.39 %
CsPT-LT-1	58,915,630	55,506,666	4,995,599,940	97.86 %	0.00 %	45.64 %
CsPT-LT-2	55,468,880	52,434,854	4,719,136,860	97.85 %	0.00 %	45.39 %
CsPT-LT-3	54,793,794	51,330,908	4,619,781,720	97.77 %	0.01 %	45.35 %
CsPT-NO-1	53,553,772	50,257,976	4,523,217,840	97.84 %	0.01 %	45.20 %
CsPT-NO-2	55,260,058	51,851,106	4,666,599,540	97.82 %	0.01 %	45.40 %
CsPT-NO-3	54,754,898	51,364,466	4,622,801,940	97.83 %	0.01 %	45.30 %

*CK* control, *LT* 4 °C treatment, *NO* NO donor DEANONOate treatment. All of the experiments were replicated three times. Q20 percentage shows the percentage of nucleotides with a quality value higher than 20; N percentage represents the percentage of unknown nucleotides in clean reads; and GC percentage indicates the percentage of guanidine and cytosine nucleotides among total nucleotides

**Table 2:** Summary of the assembly quality of the transcriptomes of the pollen tubes

	Sample	Total number	Total length(nt)	Mean length(nt)	N50	Total consensus sequences	Distinct clusters	Distinct singletons
Contig	CsPT-CK-1	60,611	20,192,162	333	571			
	CsPT-CK-2	61,246	20,491,956	335	581			
	CsPT-CK-3	63,117	20,892,307	331	561			
	CsPT-LT-1	56,102	19,226,389	343	599			
	CsPT-LT-2	58,622	19,960,405	340	595			
	CsPT-LT-3	60,547	20,132,613	333	572			
	CsPT-NO-1	59,750	19,797,938	331	559			
	CsPT-NO-2	58,884	19,734,167	335	575			
	CsPT-NO-3	59,550	19,805,943	333	568			
Unigene	CsPT-CK-1	39,726	25,741,808	648	1165	39,726	14,095	25,631
	CsPT-CK-2	40,440	26,270,780	650	1152	40,440	14,717	25,723
	CsPT-CK-3	41,626	27,483,327	660	1204	41,626	15,310	26,316
	CsPT-LT-1	36,993	24,057,930	650	1182	36,993	13,290	23,703
	CsPT-LT-2	39,070	25,937,230	664	1203	39,070	14,224	24,846
	CsPT-LT-3	39,439	25,906,326	657	1191	39,439	14,301	25,138
	CsPT-NO-1	39,514	25,545,806	647	1173	39,514	14,211	25,303
	CsPT-NO-2	38,298	24,889,732	650	1169	38,298	13,676	24,622
	CsPT-NO-3	39,061	24,971,317	639	1153	39,061	13,923	25,138
	All	45,432	46,435,079	1022	1574	45,432	22,812	22,620

Total Consensus Sequences represents all of the assembled unigenes. Distinct Clusters represents the clustered unigenes, with the same cluster containing high-similarity (more than 70 %) unigenes that may be from the same gene or homologous genes. Distinct Singletons represents unigenes from a single gene

### Annotation of predicted proteins

**Fig. 2:**
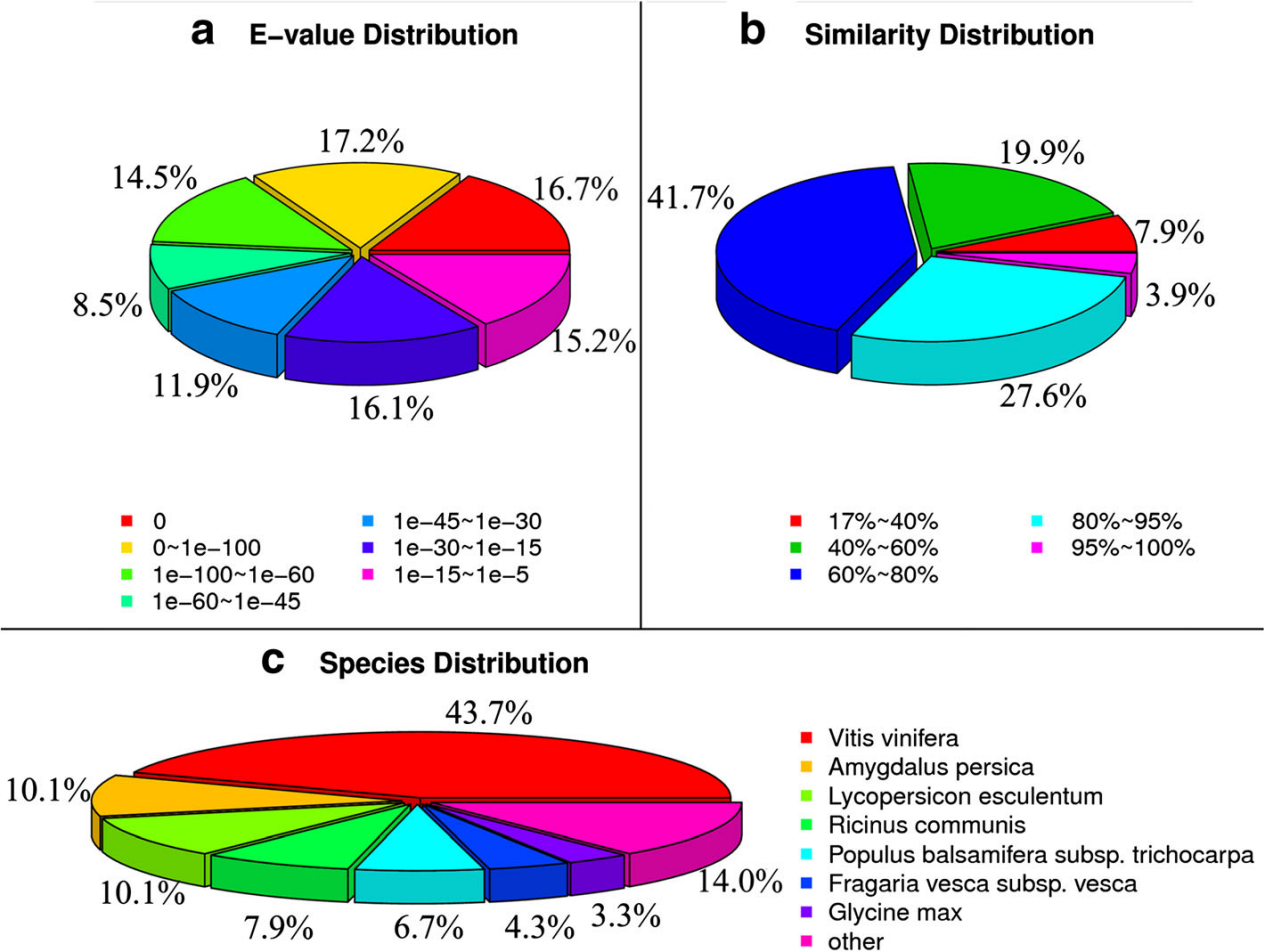
Homology search of unigenes against the NR database. **a** and **b** show the E-value and similarity distribution of the top BLAST hits for each unique sequence, respectively; **c** shows species distribution of the top BLAST hits for every homologous sequence

We annotated 36,097 unique sequences on the basis of a BLAST search of six public databases: NCBI non-redundant (NR) database, mysql-nt (NT) database, Swiss-Prot protein database, Kyoto Encyclopedia of Genes and Genomes (KEGG) database, Cluster of Orthologous Groups of proteins (COG) database and Gene Ontology (GO) database. As shown in Fig. 2, we annotated 33,716 unique sequences using the NR database as a reference. Based on these NR annotations, 48.4 % of the sequences had a very strong homology to available plant sequences (E-value < 10^−60^), 20.4 % showed a strong homology (10^−60^ < E-value < 10^−30^), and an additional 31.3 % showed homology (10^−30^ < E-value < 10^−5^) (Fig. 2a). Similarity distributions were comparable, with 31.5 % of the sequences presenting similarities higher than 80 % and 69.5 % presenting similarities of 17–80 % (Fig. 2b). Furthermore, we found 43.7 % of the sequences matching homologues from *Vitis vinifera*, with additional hits to *Amygdalus persica* (10.1 %), *Lycopersicon esculentum* (10.1 %), *Ricinus communis* (7.9 %), and *Populus balsamifera*s ubsp*. trichocarpa* (6.7 %) (Fig. 2c).

### Functional classification by COG

**Fig. 3:**
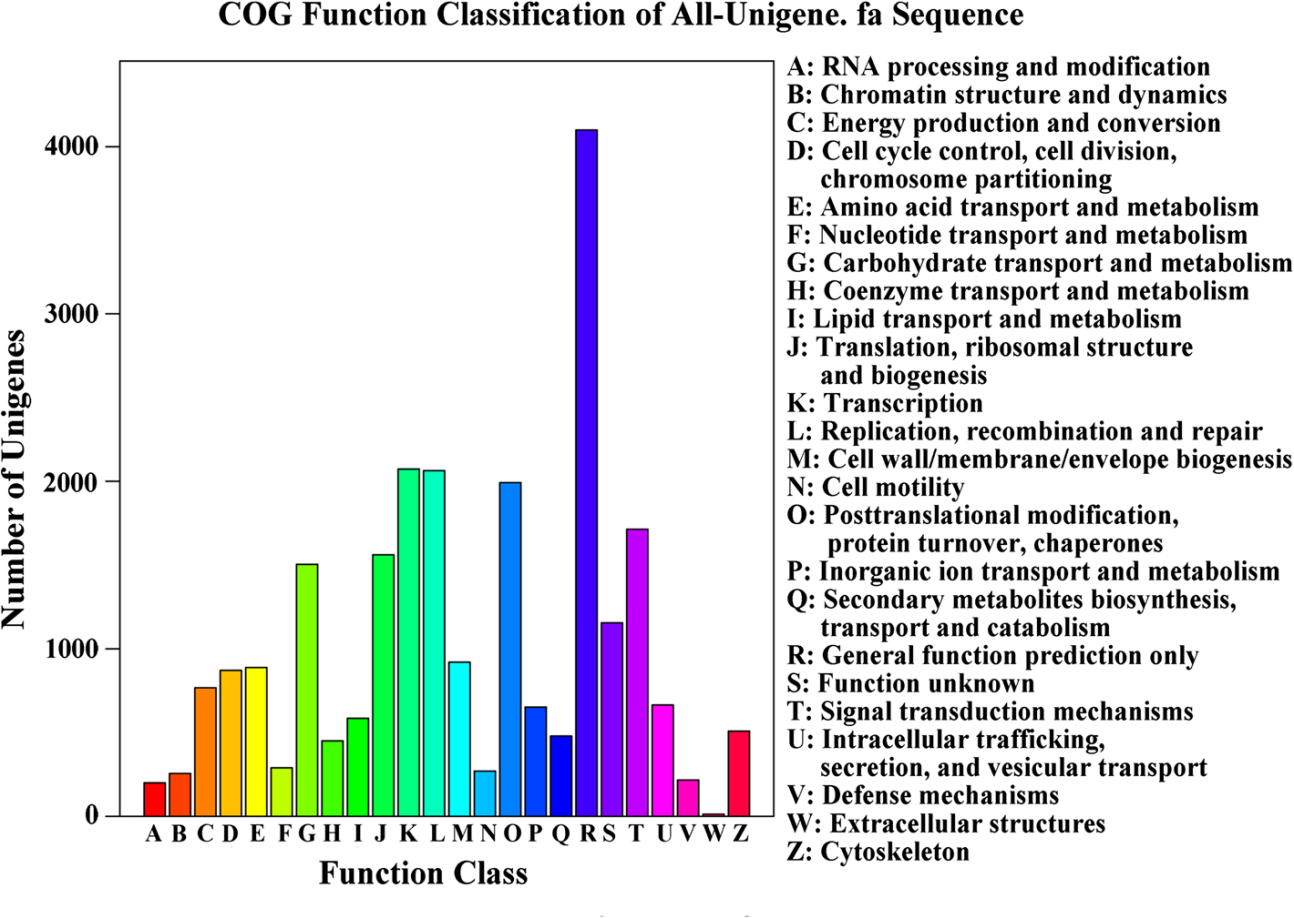
COG functional classification of the transcriptomes. Among 33,716 hits in the NR database, 13,029 unigenes with significant homologies in the COG database (E-value < 0.00001) were classified into 24 COG categories

We searched all unigenes against the COG database for functional predictions and classifications. Our query assigned 13,029 of the 33,716 sequences that presented NR hits to COG classifications (Fig. 3). The COG-annotated putative proteins were functionally classified into at least 25 molecular families, including cellular structure, biochemistry metabolism, molecular processing, and signal transduction. Among them, the cluster “General function prediction” represented the largest group (4100; 16.96 %), followed by “Transcription” (2073; 8.57 %), “Replication, recombination and repair” (2064; 8.54 %), “Posttranslational modification, protein turnover and chaperones” (1992; 8.24 %), “Signal transduction mechanisms” (1713; 7.09 %), “Translation, ribosomal structure and biogenesis” (1560; 6.45 %), and “Carbohydrate transport and metabolism” (1503; 6.22 %); Only several unigenes were assigned to “Extracellular structure”.

### DEGs and GO classification

**Fig. 4:**
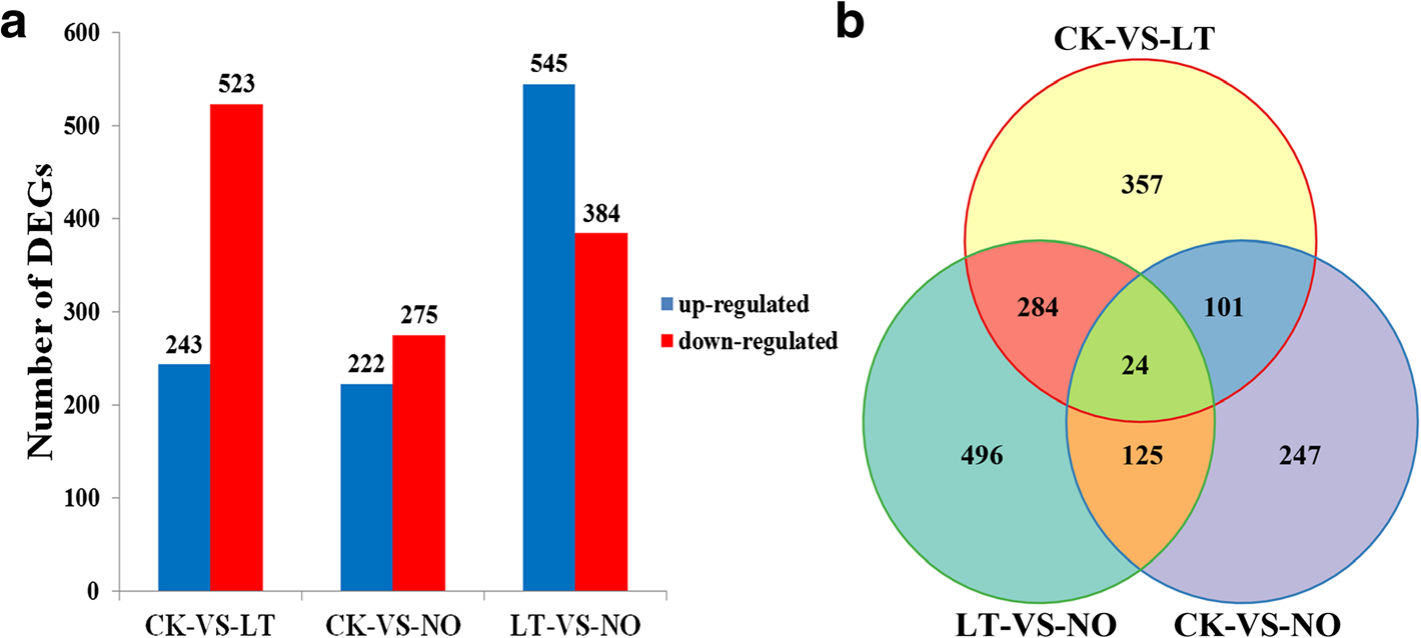
The number of DEGs identified in the comparisons of CK-VS-LT, CK-VS-NO and LT-VS-NO. CK: control; LT: low-temperature treatment; NO: NO donor DEA NONOate treatment. CK-VS-LT: comparison between CK and LT. CK-VS-NO: comparison between CK and NO. LT-VS-NO: comparison between LT and NO. The absolute values of log_2_Ratio > 1 and probability > 0.7 were used as threshold for assigning significance. **a** the number of up-regulated (*blue*) and down-regulated (*red*) DEGs in the comparisons of CK-VS-LT, CK-VS-NO and LT-VS-NO are shown, respectively. **b** the number of specifically and co-expressed DEGs from the comparisons of CK-VS-LT, CK-VS-NO and LT-VS-NO are shown, respectively

We filtered the DEGs (Additional file 2: Table S1, Additional file 3: Table S2 and Additional file 4: Table S3) using the absolute values of log_2_Ratio > 1 and probability > 0.7 as the thresholds according to Zhao et al. [31]. Comparing the library CsPT-CK with CsPT-LT (CK-VS-LT) showed that there were 766 DEGs (243 up-regulated and 523 down-regulated genes, 243/523). Moreover, we found 497 (222/275) and 929 (545/384) DEGs in the comparison between the library CsPT-CK and CsPT-NO (CK-VS-NO) and between the library CsPT-LT and CsPT-NO (LT-VS-NO), respectively (Fig. 4a). Among these DEGs, 357, 247 and 496 genes were specifically expressed in the CK-VS-LT, CK-VS-NO and LT-VS-NO, respectively. In addition, we observed 101, 284 and 125 genes co-expressed in CK-VS-LT and CK-VS-NO, CK-VS-LT and LT-VS-NO and CK-VS-NO and LT-VS-NO, respectively, and 24 genes were co-expressed in the three comparisons (Fig. 4b).

**Fig. 5:**
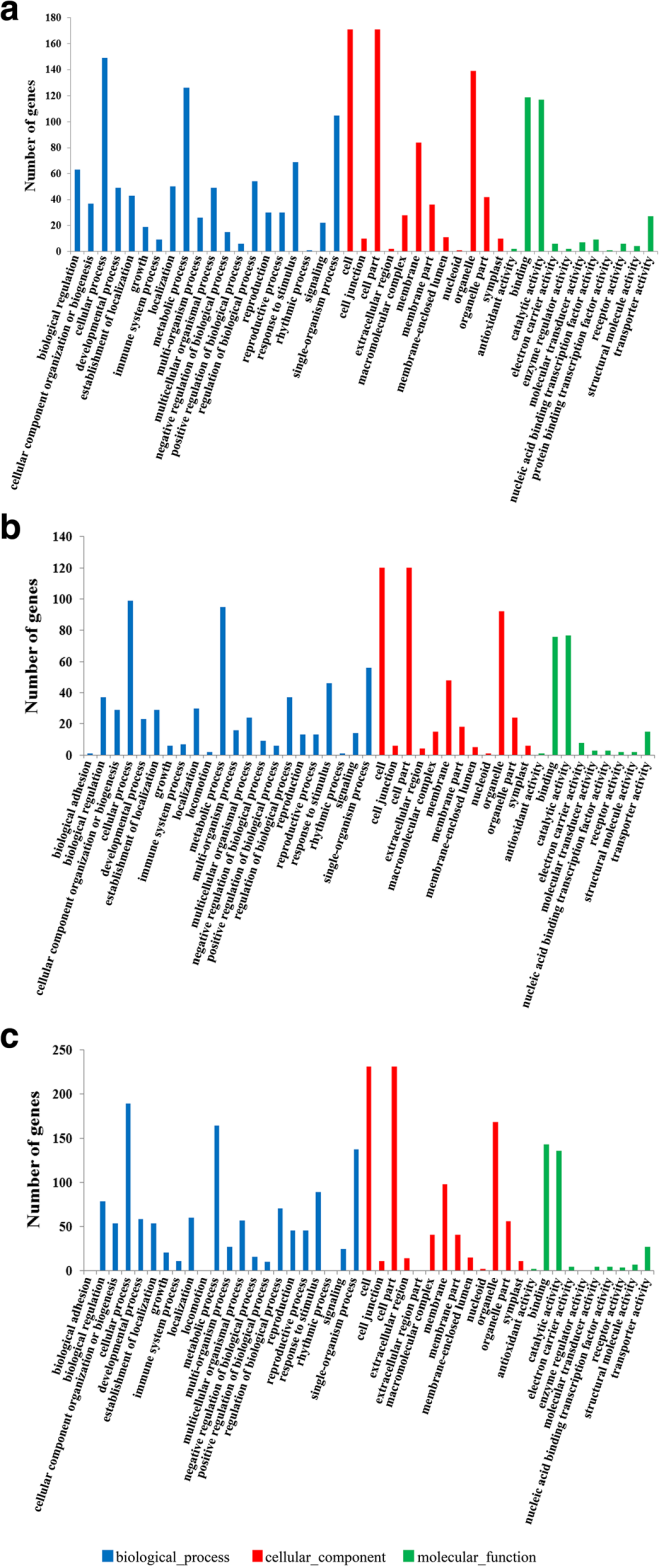
GO functional classifications of DEGs found from the comparisons of CK-VS-LT, CK-VS-NO and LT-VS-NO are annotated in the categories of Biological process (*blue*), Cellular component (*red*) and Molecular function (*green*). **a** GO classifications of DEGs from comparison of CK-VS-LT; **b**: GO classifications of DEGs from comparison of CK-VS-NO; **c**: GO classifications of DEGs from comparison of LT-VS-NO

GO classifies genes on the basis of three functional ontologies, that is, Molecular function, Cellular component and Biological process. We used Blast2GO [32] to obtain GO annotations and Web Gene Ontology Annotation Plot (WEGO) [33] to perform GO functional classifications. For the CK-VS-LT, CK-VS-NO and LT-VS-NO libraries, 262 out of 766 DEGs (262/766), 156 out of 497 DEGs (156/497) and 328 out of 929 DEGs (328/929) could be assigned a GO classification, respectively (Additional file 5: Table S4, Additional file 6: Table S5 and Additional file 7: Table S6). The 262, 156 and 328 DEGs were assigned to 1957, 1239 and 2474 GO classifications in the CK-VS-LT, CK-VS-NO and LT-VS-NO libraries, of which 952, 593 and 1219 as “Biological process”, 705, 459 and 920 as “Cellular component” and 300, 187 and335 as “Molecular function”, respectively (Fig. 5). As shown in Fig. 5, the majority of DEGs in the “Biological process” category were associated with cellular processes, metabolic processes and single-organism processes, most DEGs in the “Cellular component” category were associated with cells, cell parts and organelles, and most in the “Molecular function” category were associated with binding and catalytic activity. Furthermore, between the CK-VS-LT and CK-VS-NO libraries, certain CK-VS-LT DEGs annotated with the “Biological process” category were related to biological adhesion and locomotion, whereas several CK-VS-NO DEGs annotated with the “Molecular function” category were associated with enzyme regulator activity and protein binding transcription factor activity.

### Cluster analysis of DEGs co-expressed in CK-VS-LT and CK-VS-NO

**Fig. 6:**
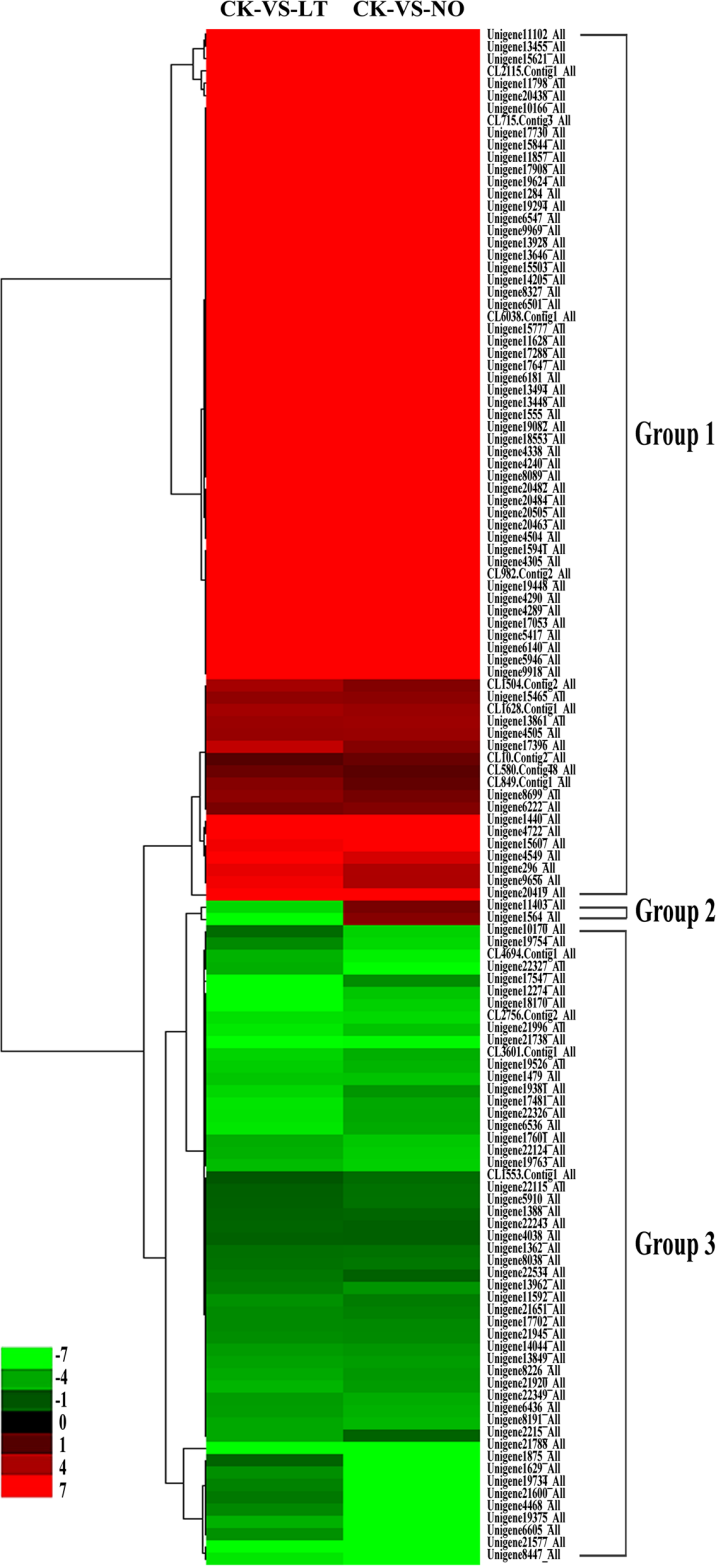
Clustering analysis of the DEGs co-expressed in CK-VS-LT and CK-VS-NO. This analysis is based on the log_2_Ratio values of DEGs. Columns displays the two comparisons (CK-VS-LT and CK-VS-NO), and rows represent the 125 DEGs. Up- and down-regulated gene expressions are showed by *red* and *green*, respectively. The 125 genes exhibit three expression patterns

Genes are usually functionally correlated to those with similar expression patterns. In order to understand the expression patterns of the 125 co-expressed DEGs (Additional file 8: Table S7) in the CK-VS-LT and CK-VS-NO libraries, a cluster analysis of the gene expression patterns between these two libraries were performed. We classified these genes into three groups (Fig. 6). The largest group (Group 1) contained 71 genes (56.8 %) (from Unigene 11102_All to Unigene 20419_All) (Additional file 8: Table S7) that were up-regulated in both CK-VS-LT and CK-VS-NO. This group mainly contained genes encoding proteins related to oxidation-reduction reactions and amino acid metabolism, such as cytochrome c oxidase, arginine/serine-rich protein and serine/threonine-protein kinase. The second largest group (Group 3) included 52 genes (41.6 %) (from Unigene 10170_All to Unigene 8447_All) that were down-regulated in both CK-VS-LT and CK-VS-NO. This group mainly contained genes encoding proteins related to plant hormone metabolism and signaling pathways and TFs, such as ethylene receptor, ABA 8’-hydroxylase (ABA8ox), MADS-box protein and CaM-binding transcription activator (CAMTA). Finally, two genes (Group 2) were down-regulated in CK-VS-LT but up-regulated in CK-VS-NO, and one of them (Unigene 11403_All) was annotated as a U-box domain-containing protein.

### DEGs identified from pollen tubes treated with low temperature and NO

In this study, numerous genes were observed that exhibited changed expression levels when pollen tubes were exposed to low temperature or NO. These genes included factors related to plant hormone metabolism and signaling pathways, such as ABA8ox and type 2C protein phosphatase (PP2C) which participate in the ABA metabolism and signaling network (Tables 3 and 4); TFs, such as MYB and ZFP transcription factors (Tables 3 and 4); vesicle polarized trafficking and cell wall biosynthesis (Additional file 9: Table S8 and Additional file 10: Table S9); the ubiquitination machinery of the ubiquitin system (Additional file 11: Table S10 and Additional file 12: Table S11); and species-specific secondary metabolite pathways (Additional file 13: Table S12 and Additional file 14: Table S13). Furthermore, many of these DEGs responded to both low temperature and NO.

**Table 3:** DEGs related to plant hormone metabolism and signaling pathways and transcription factors (TFs) between CK and LT (CK-VS-LT)

GeneID	Gene length	log_2_Ratio(LT/CK)	Up-Down-Regulation(LT/CK)	Probability	Gene annotation
Unigene14044_All	256	−1.843769497	down	0.716541745	abscisic acid 8'-hydroxylase
CL580.Contig48_All	754	1.27259979	up	0.774663281	PP2C
Unigene9712_All	720	−1.700722725	down	0.762634179	brassinosteroid insensitive 1
Unigene4038_All	663	−1.15134991	down	0.740396494	brassinosteroid insensitive 1
CL4145.Contig1_All	436	−1.824955819	down	0.800581069	BRs 1-associated receptor kinase
Unigene4486_All	236	−2.751491338	down	0.84984985	Ethylene response sensor 1
Unigene22534_All	230	−1.419906337	down	0.738457156	Ethylene response sensor 1
CL2610.Contig1_All	1080	−1.518999731	down	0.801879115	EIN3-binding F-box protein
Unigene10222_All	255	−2.230648476	down	0.7352979	EIN3-binding F-box protein
CL2941.Contig1_All	543	1.596587443	up	0.813476078	auxin-repressed protein
CL2941.Contig2_All	1062	1.516730948	up	0.810269619	auxin-repressed protein
Unigene7793_All	238	1.145845871	up	0.773991064	Auxin response factor
Unigene13496_All	379	1.090218234	up	0.761820357	indole-3-acetic acid-amido synthetase
Unigene21651_All	201	−1.631827096	down	0.734695671	indole-3-acetic acid-induced protein
Unigene15959_All	391	−1.60647149	down	0.739220521	DELLA protein
Unigene10398_All	456	1.173251983	up	0.732937816	DELLA protein
Unigene11055_All	1276	−1.283576382	down	0.760756692	Gibberellin 2 oxidase
Unigene1092_All	683	−1.991364704	down	0.737188408	Gibberellin 20 oxidase
Unigene9129_All	989	1.748223423	up	0.822590884	Gibberellin receptor GID1
Unigene21715_All	207	−5.94952025	down	0.749639884	MYB transcription factor
Unigene17702_All	299	−1.640960381	down	0.738882785	MYB transcription factor
Unigene5788_All	609	−2.816635393	down	0.906141914	Zinc finger protein
Unigene21920_All	236	−2.145887308	down	0.748960342	Zinc finger protein
Unigene9475_All	382	1.147076601	up	0.709884681	Zinc finger protein
CL1123.Contig1_All	1049	−1.09309355	0.716326082	down	MADS-box transcription factor
Unigene19750_All	364	1.683447547	0.796843185	up	MADS-box transcription factor
Unigene721_All	691	−1.099964625	0.762959708	down	MADS-box transcription factor
Unigene7926_All	400	1.064932847	0.711558713	up	MADS-box transcription factor
CL1553.Contig1_All	531	−1.026923868	0.758009229	down	MADS-box transcription factor
Unigene2215_All	492	−1.992582568	down	0.704653434	CAMTA
Unigene1182_All	690	−4.091560517	down	0.945819804	ERF transcription factor
Unigene8478_All	257	1.323055163	up	0.787730006	ERF transcription factor
Unigene7269_All	1007	−1.603627707	down	0.77292333	WRKY transcription factor
Unigene22002_All	292	−2.613167976	0.704958617	down	bHLH transcription factor
Unigene1490_All	285	−1.774205267	0.79511951	down	bHLH transcription factor

The absolute values of the log_2_Ratio (LT/CK) > 1 and probability > 0.7 were used as threshold for assigning significance. *CK* control, *LT* 4 °C treatment

**Table 4:** DEGs involved in the plant hormone metabolism and signaling pathways and transcription factors (TFs) between CK and NO (CK-VS-NO)

GeneID	Gene length	log_2_Ratio(NO/CK)	Up-Down-Regulation(NO/CK)	Probability	Gene annotation
Unigene14044_All	256	−1.843769497	down	0.716541745	abscisic acid 8'-hydroxylase
CL580.Contig48_All	754	1.070746844	up	0.727803027	PP2C
Unigene4038_All	663	−1.137884213	down	0.84974181	Leucine-rich repeat (LRR) protein
Unigene22534_All	230	−1.154935001	down	0.706870123	Ethylene response sensor 1
Unigene17743_All	272	1.415347606	up	0.7430468	indole-3-acetic acid-amido synthetase
Unigene21651_All	201	−1.525739727	down	0.723650002	indole-3-acetic acid-induced protein
Unigene17702_All	299	−1.618842865	down	0.747241362	MYB transcription factor
Unigene4344_All	557	−1.108187448	down	0.725592533	MYB transcription factor
Unigene21920_All	236	−1.849780074	down	0.72892945	Zinc finger protein
CL1553.Contig1_All	531	−1.28555	up	0.738229	MADS-box transcription factor
Unigene1516_All	230	1.242887153	up	0.792390331	CAMTA
Unigene2215_All	492	−1.171631887	down	0.701307237	CAMTA
Unigene20631_All	228	−5.122748755	down	0.78094284	ERF transcription factor
Unigene13943_All	358	−1.523794549	down	0.785566632	bHLH transcription factor
CL898.Contig1_All	231	−2.128187976	down	0.744496571	bHLH transcription factor

The absolute values of log_2_Ratio (NO/CK) > 1 and probability > 0.7 were used as threshold for assigning significance. *CK* control, *NO* NO treatment

### Quantitative real-time PCR (qRT-PCR) validation of DEGs in CK-VS-LT and CK-VS-NO

**Fig. 7:**
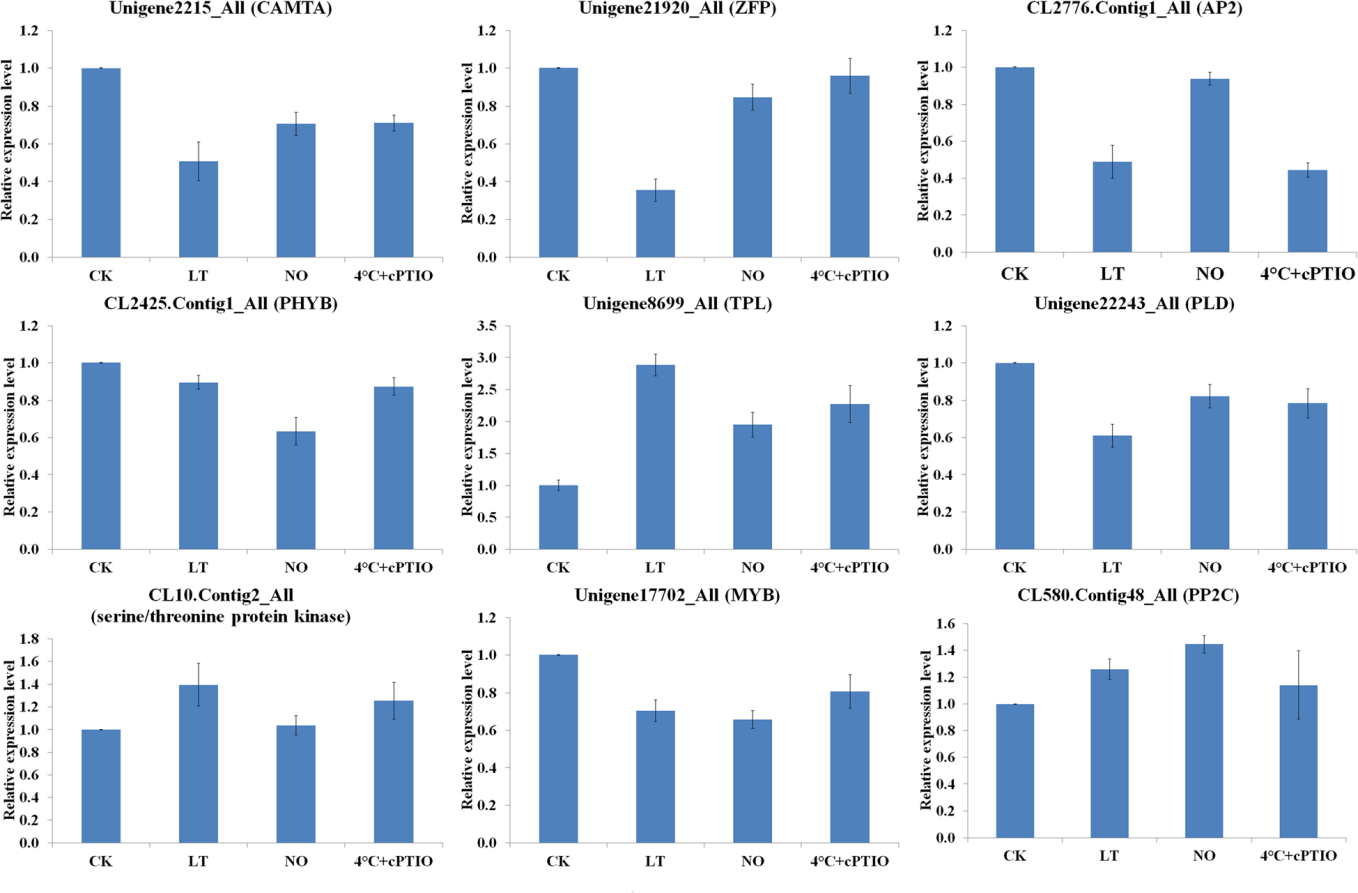
QRT-PCR analysis confirms the expression patterns indicated by the sequencing. Nine genes were randomly selected for qRT-PCR analysis and their primers were presented in Additional file 16: Table S14. The *C. sinensis* 18S rRNA was employed as reference

To confirm the gene expression patterns shown in the sequencing data, we performed a qRT-PCR analysis on nine randomly selected genes in pollen tubes exposed to low-temperature stress, 25 μM DEA NONOate (NO donor) or to low temperature with 200 μM 2-(4-carboxyphenyl)-4,4,5,5-tetramethylimidazoline-1-oxyl-3-oxide (cPTIO) (NO scavenger). According to our preliminary analysis and previous reports, we speculated that Ca^2+^ signaling and TFs played an important role in this process [11, 25]. Therefore, we selected CAMTA (Unigenes 2215), MYB (Unigenes 17702), ZFP (Unigenes 21920), AP2 (CL2776.Contig1) and PP2C (CL580.Contig48) genes. Furthermore, Phospholipase D (PLD) accumulated highly when *Chorispora bungeana* was exposed to low temperature [34] and PLD activation in *C. bungeana* exposed to low-temperature stress was related to a drop in Ca^2+^ content in membrane fractions [35]. Moreover, TOPLESS (TPL) was involved in mutual adjustment between hormones via NO in *Petunia hybrida* [36]. Additionally, genes encoding a non-specific serine/threonine protein kinase were down-regulated expressed during response to low temperature in two grapevine varieties [37]. Recent research indicated that phytochrome B (PHYB) and PHYA antagonistically regulated tomato low-temperature tolerance, in a process involving far-red light-induced PHYA activation to induce ABA signaling and eventually lead to cold response [38]. Here, we also randomly selected PLD (Unigenes 22243), TPL (Unigenes 8699) and serine/threonine protein kinase (CL10.Contig2) in CK-VS-LT and CK-VS-NO, and the PHYB (CL2425.Contig1) in CK-VS-LT for our qRT-PCR assay. The expression of Unigene 8699, CL10.Contig2 and CL580.Contig48 was up-regulated, whereas Unigenes 2215, 17702, 21920 and 22243 and CL2425.Contig1 and CL2776.Contig1 were down-regulated expressed in CK-VS-LT and CK-VS-NO (Fig. 7). These analyses support our DEGs data, and the high confirmation rate further confirmed the reliability of the data. In addition, application of 200 μM cPTIO under low-temperature stress rescued the changes in the expression levels of the DEGs co-expressed in the three treatments, such as Unigenes 2215, 8699, 17702, 21920 and 22243; CL10.Contig2 and CL580.Contig48. These results indicated that the seven genes intervened in pollen tube response to low temperature, possibly via the regulation of NO signaling.

## Discussion

According to our previous report, NO negatively regulated *C. sinensis* pollen tube growth under low-temperature stress partially through the cGMP signaling pathway [11]. However, the potential molecular mechanism during this process is still unknown. Here, we comprehensively analyzed the gene expression changes in *C. sinensis* pollen tubes treated with low temperature and exogenous NO by RNA-Seq technology to investigate the pollen tube’s defense response to low temperature. Genes related to metabolism and signaling pathways of plant hormones, TFs, vesicle polarized trafficking and cell wall biosynthesis, the ubiquitination machinery of the ubiquitin system and species-specific secondary metabolite pathways were the main focus of this study.

### Comparison of unigenes between *C. sinensis* leaves and pollen tubes

According to previous reports, Illumina sequencing of leaves transcriptome from *C. sinensis* cv. ‘Longjing43’ revealed an average transcript size of 356 bp with a N50 of 529 bp [5]. In this study, we reported that the average transcript size was > 331 bp, and the N50 was > 559 bp in every transcriptome of pollen tubes from *C. sinensis* cv. ‘Longjingchangye’. In addition, the previous study showed the annotation of 53,201 unigenes based on BLASTx searches against NR (51,883 unigenes), UniRef90 (52,217 unigenes), the Arabidopsis Information Resource (TAIR, version 10) (42,969 unigenes), KEGG (15,086 unigenes) and COG (29,356 unigenes) databases in tea plant leaves [5]. Among 71,289 transcripts based on NR annotation, 30,115 transcripts matched 80,176 GO term annotations [5]. Most of the unigenes both from leaves and pollen tubes are categorized as the cell and cell part (cellular component), binding and catalytic activity (molecular function) and cellular process and metabolic process (biological process) (Additional file 15: Figure S2). Interestingly, envelope (cellular component), auxiliary transport protein, transcription regulator (molecular function) and anatomical structure formation, cell killing, death, pigmentation, viral reproduction (biological process) were only annotated in GO categories of tea leaves transcriptome (Additional file 15: Figure S2A). However, cell junction, extracellular matrix, extracellular matrix part, membrane, membrane part, nucleoid, symplast (cellular component), channel regulator, metallochaperone, nucleic acid binding transcription factor, nutrient reservoir, protein binding transcription factor, protein tag, receptor (molecular function) and regulation of biological (biological process) were only annotated in GO categories of pollen tube transcriptome (Additional file 15: Figure S2B). These differences between transcriptomes from leaves and pollen tubes might result from the diversity of reproductive and vegetative tissues of plant [6], further confirming that reproductive tissue responds to low temperature stress using distinct mechanisms from vegetative tissues [7].

### Plant hormone metabolism and signaling pathways

Plant hormones play essential roles in response to abiotic stress through their involvement in complicated signaling networks and physiological processes. For example, ABA has been shown to accumulate as a protective factor against cold injury in maize [39], and previous investigations have demonstrated that NO enhances tolerance to drought stress via promoting ABA-induced stomata closure [40, 41]. In our study, we identified one *ABA8ox* gene (Unigene14044_All) and one *PP2C* gene (CL580.Contig48_All) in the CK-VS-LT (Table 3) and CK-VS-NO libraries (Table 4). As shown in previous reports, NO-induced ABA content reduction in *Arabidopsis* correlates with the modulation of ABA8ox protein expression [42]. Additionally, ABA signaling is negatively regulated by NO in germination and early seedling growth in *Arabidopsis* through the S-nitrosylation of sucrose non-fermenting 1 (SNF1)-related protein kinase 2.2 (SnRK2.2) and SnRK2.3 [43]. As a negative regulator, PP2C inactivates SnRK2, thereby regulating ABA signals [44]. These reports and our studies indicate that NO might participate in ABA catabolism and signaling to regulate pollen tube elongation under cold stress through the genes *ABA8ox* (Unigene14044_All) and *PP2C* (CL580.Contig48_All). In addition, the plant steroidal hormone BRs has been found to promote growth and cell elongation and protect plants against abiotic stresses. Reports have shown that BRs exert positive effects on the mitigation of oxidative damage by enhancing the antioxidant defense system in *Chorispora bungeana* under cold stress [45]. BRs are recognized through an active complex of a leucine-rich repeat (LRR) receptor-like kinase (RLK) brassinosteroid-insensitive 1 (BRI1) and BRI1-associated kinase 1 (BAK1). BRs bind to the extracellular LRR domain of BRI1 and induce a phosphorylation-mediated cascade to modulate gene expression [46]. Meanwhile, BAK1 is involved in the heterodimerization and endocytosis associating with BRI1 to promote BRs-induced signal transducing instead of involved in binding to BRs. [47]. We identified two *BRI1* genes (Unigene9712_All and Unigene4038_All) and one *BAK1* gene (CL4145.Contig1_All) in CK-VS-LT (Table 3) as well as one *BRI1* gene (Unigene4038_All) in CK-VS-NO (Table 4). Current studies have revealed that NO concentration in the root cells is increased by BRs, which is required for BR-induced alterations of the root architecture in *Arabidopsis* [48]. This finding suggests that BRs are involved in pollen tube resistance to low temperature through Unigene9712_All, Unigene4038_All and CL4145.Contig1_All and indicate that NO might participate in this process through Unigene4038_All. Moreover, BRs could overcome the inhibition of seed germination induced by ABA and increase the tolerance of *Arabidopsis* seedlings to cold [49]. BR-induced NO production and NO-activated ABA biosynthesis are significant mechanisms for BR-increased tolerance to water-stress [50]. Combining these reports and our findings, BRs and ABA might interact in NO signal transduction during the pollen tube responding to low-temperature stress, although the underlying mechanisms require further research.

Ethylene (ET) is a gaseous plant hormone, and its signaling pathways participate in plant responses to low temperature [51, 52]. Previous reports indicated that ET signaling negatively regulates *Arabidopsis* tolerance to cold stress by inhibiting the expression of *CBF* and *type-A ARR* genes [53]. Ethylene response sensor 1 (ERS1) has been identified as one of the membrane-located receptor proteins in the ET signaling pathway [54]. The level of ET is usually low in normal conditions, and its response is suppressed by ERS1 via activating the downstream negative regulator CTR1 (Constitutive Triple Response 1). Furthermore, EIN2 (ethylene insensitive 2) protein acting as a critical positive regulator of ET signaling has been found to be inhibited by CTR1 in *Arabidopsis* [55]. As downstream of EIN2, the plant-specific transcription factors EIN3 and EIN3-like1 (EIL1) are sufficient and necessary to stimulate ethylene-response genes expression and to modulate the ethylene-related responses of plants [56]. EIN3-Binding F-box-1 (EBF1) and EBF2 tightly regulate the stability of EIN3 at the protein level [57], and EBF2 transcription is in turn activated by EIN3, thereby forming a negative feedback loop [58]. Interestingly, recent work has shown that ERS1 and EBF1/2 are differentially expressed in Papaya fruit ripening disorders caused by cold injury [59]. In our study, two *ERS1* genes (Unigene4486_All and Unigene22534_All) and two *EBF1/2* genes (CL2610.Contig1_All and Unigene10222_All) were down-regulated between CK and LT (CK-VS-LT) (Table 3). These findings imply that *ERS1* and *EBF1/2* are involved in pollen tube resistance to low temperature. Although few reports on the interaction between the two gaseous hormones NO and ET during low-temperature stress are available, studies have suggested that NO and ET have a complicated relationship in plants during water logging stress [60], salt stress [61], UV-B stress [62] and hypersensitive responses [63]. One *ERS1* gene (Unigene22534_All) was also identified in the CK-VS-NO library (Table 4). It is tempting to speculate that *ERS1* (Unigene22534_All) responds to low temperature via the NO signaling pathway and the other *ERS1* genes in *C. sinensis* pollen tubes (Unigene4486_All and *EBF1/2* (CL2610.Contig1_All and Unigene10222_All) respond to low temperature in a NO-independent manner.

Auxin is an essential morphogenetic signal participating in the regulation of cell identity during all the developmental processes of plants, and auxin signaling pathways constitute a critical component of mechanisms for plant tolerance to abiotic stresses. Substantial evidence has demonstrated a tight link between auxin responses and cold stress [64, 65]. For example, cold stress affects auxin polar transport via the selective inhibition of the intracellular trafficking of numerous proteins in *Arabidopsis*, including the auxin efflux carriers [66]. Two *auxin-repressed protein* genes (CL2941.Contig1_All and CL2941.Contig2_All), one *auxin response factor* gene (Unigene7793_All), one *indole-3-acetic acid (IAA)-amido synthetase* gene (Unigene13496_All) and one *IAA-induced protein* gene (Unigene21651_All) were identified in the CK-VS-LT library in this study (Table 3). Interestingly, as shown in a recent report, auxin participates in regulating pollen tube tip growth [67]. Therefore, it is reasonable to speculate that auxin intervenes in pollen tube responding to low-temperature stress in *C. sinensis*. Furthermore, Markus et al. [68] reported that the release of IAA and NO from peroxisomes is a spatially and temporally coordinated process in root formation in maize and *Arabidopsis*, thereby indicating that auxin and NO interact closely in plant developmental processes. We identified one *IAA-amido synthetase* gene (Unigene17743_All) and one *IAA-induced protein* gene (Unigene21651_All) in CK-VS-NO (Table 4). Unigene21651_All was simultaneously detected in both CK-VS-LT and CK-VS-NO libraries, indicating that *IAA-induced protein* (Unigene21651_All) might be related to the NO-mediated pathway in *C. sinensis* pollen tube growth in responding to low-temperature stress.

Additionally, gibberellins (GAs) are another class of plant hormones that play critical roles in modulating plant growth and development in response to environmental cues. Exposure of *Arabidopsis* seedlings to cold stress triggers a reduction in bioactive GA, which restrains root growth [69]. Previous studies have shown that the nuclear-localized growth-repressing DELLA proteins are central components of the GA-signaling pathway. These proteins accumulate through a posttranslational mechanism mediated by the decrease in bioactive GA via up-regulating the transcript level of GA 2-oxidase (GA2ox) gene, and this in turn increases the transcript levels of GA20ox and GA3ox gene by a feedback mechanism [70, 71]. The binding of bioactive GA to GA insensitive dwarf 1 (GID1), a pocket of the GA receptor, promotes the interaction between GID1 and DELLAs and forms the GA–GID1–DELLA complex [72]. This model has been employed to reveal the molecular mechanisms for GA perception. In this study, two *DELLA* genes (Unigene15959_All and Unigene10398_All), one *GA2ox* gene (Unigene11055_All), one *GA20ox* gene (Unigene1092_All) and one *GID1* gene (Unigene9129_All) showed a significantly different expression in CK-VS-LT (Table 3). These findings suggest that GA participates in pollen tube response to low-temperature stress, whereas none of the GA signals or synthetic metabolism genes was detected in the DEGs of CK-VS-NO. In addition, María et al. [73] speculated that high levels of NO content could influence the increase of DELLA activity through the disappearance of PIN-FORMED 1 (PIN1) and subsequently inhibit cell elongation in the elongation-differentiation zone in *Arabidopsis*. However, the *PIN1* gene is not detected in CK-VS-NO. Therefore, we hypothesize that NO might regulate *C. sinensis* pollen tube growth during responses to low temperature stress through a GA-independent signaling network.

### Transcription factors (TFs)

Plants have evolved complex and highly efficient regulatory networks to respond and adapt to cold stress, and TFs play crucial roles in these regulatory processes. For example, the rice R2R3-type MYB gene, *OsMYB2*, plays a positive regulatory role in low-temperature stress tolerance [74]. Similarly, the soybean typical Cys2/His2-type (C2H2-type) zinc finger *GmZF1* enhances cold stress tolerance by regulating gene expression in transgenic *Arabidopsis* [75]. In our study, two *MYB* genes and three *ZFP* genes were identified in CK-VS-LT as shown in Table 3. The results reveal that MYB and ZFP are involved in pollen tube elongation in response to low-temperature stress. Besides, NO action inhibits the DNA-binding of AtMYB2 through the S-nitrosylation of Cys53 [76]. Furthermore, the thiols of zinc-sulfur clusters can be S-nitrosated by NO, which reversibly disrupts zinc finger structures and presents a molecular mechanism for regulating ZFP TFs [77]. One *MYB* gene (Unigene17702_All) and one *ZFP* gene (Unigene21920_All) were detected among the comparisons of the CK-VS-LT and CK-VS-NO libraries (Tables 3 and 4), implying that *MYB* (Unigene17702_All) and *ZFP* (Unigene21920_All) are related to the NO signaling pathway during low-temperature stress responses in *C. sinensis* pollen tubes. Moreover, NO negatively regulates the DNA binding of MYB2 by S-nitrosylation in the ABA-dependent cold signaling pathway [25]. Combining this information with the results for ABA and NO, we speculate that *MYB* (Unigene17702_All) might participate in *C. sinensis* pollen tube growth during low-temperature stress via an ABA-dependent pathway that acts as a NO signaling pathway downstream regulator.

Pollen tube elongation is a tip growing process that can be affected by TFs. For example, the pollen-specific MIKC* class of MADS-box TFs is necessary for pollen maturation and tube elongation and controls a transcriptional switch that directs pollen maturation in *Arabidopsis* [78]. Furthermore, a large number of reports reveal that calmodulin (CaM) is a key element in pollen tube elongation and orientation and dependent on a complicated crosstalk process between multiple pathways [79–81]. The CaM-interacting protein group CAMTA has been identified in the plant defense signaling cascade [82]. Moreover, MADS-box [83] and CAMTA [84] TFs take part in the regulation of plant responses to low temperature. We detected five *MADS-box* genes and one *CAMTA* gene in CK-VS-LT (Table 3). These studies and our results indicate that CAMTA and MADS-box TFs participate in pollen tube elongation under cold stress. In CK-VS-NO, one *MADS* gene and two *CAMTA* genes were also detected, and in CK-VS-LT and CK-VS-NO, one *MADS* gene (CL1553.Contig1_All) and one *CAMTA* gene (Unigene2215_All) were differentially expressed (Tables 3 and 4). Previous experiments have demonstrated that NO modulates Ca^2+^ signaling in lily and *Arabidopsis* pollen tubes through directly imaging the concentration of cytosolic free Ca^2+^ during NO-induced tube re-orientation [26, 27]. We speculate that the *CAMTA* gene (Unigene2215_All) might participate in the regulation of Ca^2+^ by NO during pollen tube elongation under cold stress. Additionally, although directly relevant reports are not available on the crosstalk between MADS-box and NO during pollen tube growth, arginine is the substrate of NO synthases [85], and the metabolism of arginine is regulated by MADS-box [86]. Thus, it is tempting to suggest that the *MADS* gene (CL1553.Contig1_All) might influence *C. sinensis* pollen tube elongation under low temperature via the NO synthesis pathway.

In addition to the above TFs, we also identified two *ERF* genes, one *WRKY* gene and two *bHLH* genes in CK-VS-LT (Table 3) as well as one *ERF* gene and two *bHLH* genes in CK-VS-NO (Table 4). Using forward and reverse genetics, the ERF [51], WRKY [87] and bHLH [88] TFs were isolated and identified as the molecular switches of cold-responsive signaling networks in plants. Although these three TF family genes were found in CK-VS-LT and CK-VS-NO, none were simultaneously found in both CK-VS-LT and CK-VS-NO libraries. Thus, the complex networks and mechanisms of NO, ERF, WRKY, bHLH and low-temperature stress require further research.

### Vesicle polarized trafficking and cell wall biosynthesis related genes

Pollen tube growth is a highly polarized process which is dependent on the polarized trafficking of vesicles containing cell wall materials to the tip region to establish a restricted growth zone [89]. Previous reports have shown that secretory vesicle formation in root hairs is impaired in the double phosphatidylinositol-4-kinase (PI4K) *Arabidopsis* mutant *pi4kβ1/β2* because of the disruptions to phosphatidylinositol-4-phosphate (PI4P) gradient and polarized vesicle trafficking [90]. Furthermore, PI 4-phosphate 5-kinase (PIP5K) isoforms can catalyze the PI 4, 5-bisphosphate (PIP2) synthesis, which eventually regulates vesicle trafficking [91]. Additionally, Feng et al. [92] reported that vesicle fusion and recycling in the tip region of pollen tube apex might be mediated by vesicle-associated membrane protein 726 (VAMP726) in *Petunia inflata*. Reports have indicated that the vesicle trafficking process is accelerated in response to low temperature stress in *Arabidopsis* [93]. Here, we identified four *PI4K* genes, one *PIP5K* gene and two *VAMP* genes in CK-VS-LT (Additional file 9: Table S8), implying that these genes modulate pollen tube elongation during the response to cold stress. We also found that one of the *PI4K* genes (Unigene13861_All) is also differentially expressed in CK-VS-NO (Additional file 10: Table S9). Interestingly, NO involvement in pollen tube growth regulation has been reported to occur via the modulation of vesicle polarized trafficking [29], and NO is also required for vesicle formation and trafficking in root hair tip growth in *Arabidopsis* [94]. These results indicate that NO may modulate *C. sinensis* pollen tube elongation under low-temperature stress by mediating vesicle formation and trafficking via the *PI4K* gene (Unigene13861_All). Additionally, cell wall construction is among the most important mechanisms in pollen tube tip growth [95] and could be regulated by NO in *Pinus bungeana* [29]. COBRA-like genes are important factors for secondary cell wall development in the root growth zone of *Arabidopsis* [96]. We also identified one *COBRA-like* gene (Unigene10170_All) in the DEGs between CK-VS-LT and CK-VS-NO (Additional file 9: Table S8 and Additional file 10: Table S9). This *COBRA-like* gene (Unigene10170_All) might participate in cell wall construction in *C. sinensis* pollen tubes during low temperature stress through the NO signaling pathway.

### Ubiquitination machinery of the ubiquitin system

The protein ubiquitination pathway is one of the most effective processes for performing versatile post-translational modifications that mediate the growth and development of all eukaryotic species. Ubiquitylated proteins are targeted for degradation by the 26S proteasome which is a proteolytic machine that degrades the target and allows ubiquitin moieties to be reused. Ubiquitin–26S proteasome system (UPS) widely intervenes in plant growth and development during tolerance to stresses by modulating proteins activity or affecting their localization or stability [97]. The UPS depends on the activity of ubiquitin activating enzymes (E1s), ubiquitin conjugating enzymes (E2s) and ubiquitin ligases (E3s). E3s are recognized as the critical factors that define substrate specificity [98] and are divided into four major types: Really Interesting New Gene (RING)-type, cullin–RING ligases (CRLs), Homology to E6-Associated Carboxyl-Terminus (HECT)-type and U-box-type. Furthermore, U-box-type E3s transfer ubiquitin tags directly from E2s-Ubs to target proteins [99]. Additionally, CRLs can consist of a substrate-recruiting protein, such as F-box [100] and broad complex, tramtrack, bric-a-brac/pox virus and zinc finger (BTB/POZ) [101]. In our study, we identified one *E2* gene, four *E3* genes, one *26S proteasome* gene, four *U-box* genes, nine *F-box* genes and three *BTB/POZ* genes in the CK-VS-LT library (Additional file 11: Table S10). UPS modulates the levels of stress hormone biosynthesis and secondary messengers as well as the abundance of regulatory proteins that may be accumulated resulting from exposure to abiotic stress [102], which implies that the UPS plays a fundamental role in *C. sinensis* pollen tube elongation under low temperature stress. Moreover, most E3s have been linked to abiotic stress responses through their modulation of various stress signaling processes [98]. Previous reports have shown that multiple E3s regulate ABA-dependent stress signaling [103]. Combined with our findings on ABA and cold stress, this information indicates that the UPS is involved in an ABA-dependent signaling pathway and might influence other hormones and TFs through ABA-dependent signaling. Additionally, certain genes encoding E3 proteins are involved in NO-dependent modulation in response to cryptogein in *Arabidopsis* [104], indicating that NO and UPS interact closely in plants. In CK-VS-NO, four *E3* genes, one *26S proteasome* gene, two *U-box* genes, one *F-box* gene and one *BTB/POZ* protein gene were identified (Additional file 12: Table S11). Additionally, we also identified one *E3s* (Unigene6536_All), one *26S proteasome* (Unigene21945_All), one *U-box* (Unigene11403_All) and one *BTB/POZ* (Unigene17396_All) gene co-expressed in the DEGs of CK-VS-LT and CK-VS-NO. These results indicate that the *E3s* (Unigene6536_All), *26S proteasome* (Unigene21945_All), *U-box* (Unigene11403_All) and *BTB/POZ* (Unigene17396_All) might participate in an NO-dependent signaling pathway in *C. sinensis* pollen tubes under low temperature stress through their influence on the modulation of the UPS, which alters the regulatory proteins levels.

### Species-specific secondary metabolites pathways

Major secondary metabolites are fundamental components that define the flavor, taste and health benefits of tea, such as flavonoids, caffeine and theanine. Flavonoids contain flavones, flavonols, isoflavones, flavanones, flavanols, and anthocyanidins, which have various functions in plant tolerance to stresses, including defense against phytopathogens, protection from ultraviolet light and antioxidant activity [105]. Furthermore, the accumulation of catechins in *C. sinensis* is very susceptible to low temperature [106]. In addition, caffeine is a purine alkaloid widely used as stimulant and pharmaceutical component, and mainly synthesized in young leaves in *C. sinensis* via a typical biosynthetic pathway that includes steps of purine synthesis and modification [107]. Theanine is emerged as a unique free amino acid that confers the unique “umami” taste of tea, and it occupies about half of the total free amino acids in tea [108]. It has been revealed that theanine biosynthesis starts from glutamine and pyruvate and includes further synthetic steps in buds, leaves and roots from *C. sinensis* [109, 110]. In the present study, some genes related to flavonoids, caffeine and theanine biosynthesis pathways were down-regulated in CK-VS-LT (Additional file 13: Table S12). In addition, we also identified some genes in CK-VS-NO (Additional file 14: Table S13). And one *caffeine synthase 1* (Unigene1362_All) and *glutamate receptor* gene (CL4694.Contig1_All) were co-expressed in the comparisons of CK-VS-LT and CK-VS-NO. These results reveal that caffeine and theanine mediate cold stress tolerance via NO signal pathway by *caffeine synthase 1* (Unigene1362_All) and *glutamate receptor* (CL4694.Contig1_All).

## Conclusions

**Fig. 8:**
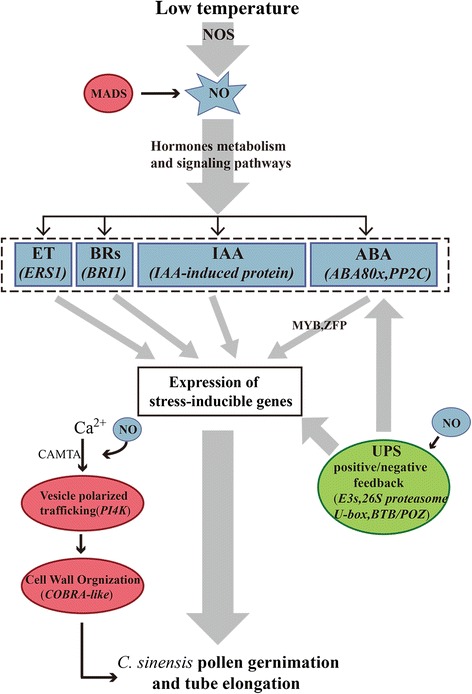
Model of the potential signaling pathway of NO in *C. sinensis* pollen tube response to low temperature. This summarized model was based on the results reported by Wang et al. [111] and Lyzenga *ea al*. [102]. Plant hormone signaling pathways (ABA, BRs, ET and auxin) respond to pollen germination and tube elongation at low temperature, and then TFs (MYB, ZFP, CAMTA and MADS-box) modulated relevant proteins expression. Moreover, the UPS (E3s, 26S proteasome, U-box and BTB/POZ) probably involves in affecting pollen germination and tube growth in response to low-temperature stress by modulating the activity, localization or stability of different proteins. Additionally, Ca^2+^ gradient, vesicle polarized trafficking as well as cell wall biosynthesis might participate in pollen tube elongation under low-temperature stress through the NO signaling pathway by CAMTA, PI4K and COBRA-like

We produced a dataset containing 45,432 unigenes from the *C. sinensis* pollen tube transcriptomes using paired-end and *de novo* sequencing with the Illumina HiSeq™2000 platform. Among these unigenes, 36,097 were annotated with descriptions from the NR, NT, Swiss-Prot, KEGG, COG and GO databases. Our dataset includes comprehensive analysis in sequence and DEGs profiling data that provide a dynamic perspective on transcriptomic variations caused by NO and low-temperature in *C. sinensis* pollen tube elongation. Many DEGs were simultaneously found in both the CK-VS-LT and CK-VS-NO libraries in different functional pathways, such as plant hormone signaling (ABA, BRs, ET and auxin), TFs (MYB, ZFP, CAMTA and MADS-box), vesicle polarized trafficking and cell wall biosynthesis (PI4K and COBRA-like), the ubiquitination machinery of the ubiquitin system (E3s, 26S proteasome, U-box and BTB/POZ) and caffeine and theanine biosynthesis (caffeine synthase and glutamate receptor). Combining our findings and previous reports, we propose a crosstalk network which may indicate the probable mechanisms underlying NO mediating *C. sinensis* pollen tube growth during the response to low-temperature stress (Fig. 8) [102, 111]. Plant hormone signaling pathways (ABA, BRs, ET and auxin) respond to pollen germination and tube elongation at low temperature, and then TFs (MYB, ZFP, CAMTA and MADS-box) modulated relevant proteins expression. Moreover, the UPS (E3s, 26S proteasome, U-box and BTB/POZ) probably involves in affecting pollen germination and tube growth in response to low-temperature stress by modulating the activity, localization or stability of different proteins. Additionally, Ca^2+^ gradient, vesicle polarized trafficking as well as cell wall biosynthesis might participate in pollen tube elongation under low-temperature stress through the NO signaling pathway by CAMTA, PI4K and COBRA-like. Taken together, these findings offer a more profound understanding of the candidate genes employed by *C. sinensis* pollen tubes to integrate low temperature signals and finely tune their tolerance responses, present a fundamental transcriptomic resource to further explore the molecular mechanisms of reproductive tissue tolerance to low-temperature.

## Methods

### Plant materials

Mature pollen was collected from *C. sinensis* (L.) O. Kuntze cv. ‘Longjingchangye’ growing in Dr. Sun Yat-sen’s Mausoleum Tea Factory, Nanjing, China (http://www.yhc1958.com/index.html). Then the pollen was pre-cultured in a control liquid medium (5 % sucrose, 0.05 % Ca(NO_3_)_2_ **·** 4H_2_O, 0.01 % H_3_BO_3_, 5 % PEG 4000 and 30 mM MES, pH 6.0) at 25 °C in darkness for 30 min [111]. The pre-cultured samples were incubated at 25 °C for 1 h as control (CK treatment). The pre-cultured pollen was incubated at 4 °C for 1 h as low-temperature treatment (LT treatment). In addition, adding 25 μM NO donor DEA NONOate to the pre-cultured pollen and incubated it at 25 °C for 1 h as NO treatment (NO treatment). For NO scavenger treatment, the pre-cultured pollen was incubated in the medium with 200 μM cPTIO at 4 °C for 1 h (LT with cPTIO treatment). All experiments were replicated three times. Pollen tubes snap frozen in liquid nitrogen after collection and then stored at −80 °C before use.

### RNA extraction, library preparation and RNA-Seq

Nine pollen tube samples from three independent experiments were collected after incubated under control, low temperature and NO treatment. For Illumina sequencing, total RNA was extracted and treated with RNAiso Plus (TaKaRa, Japan) and RNase-free DNase I (Takara Biotechnology, China), respectively, according to the manufacturer’s instructions. We confirmed the integrity, quality and quantity of total RNA using Agilent 2100 Bioanalyzer (Agilent, Santa Clara, CA, USA) and a spectrophotometer (NanoDrop, Wilmington, DE), and used TruSeq RNA Sample Prep Kit v2 (Illumina) to purify 200 ng total RNA for each sample by oligo-dT. Then the total RNA was fragmented using Elute, Prime and fragmentation buffer. We produced the first-strand cDNA using First Strand Master Mix and Super Script II (Invitrogen) and created the second-strand cDNA using Second Strand Master Mix. The double-stranded DNA was then purified by QiaQuik PCR purification kit (Qiagen, Beijing, China) before end repaired by incubation with End Repair Mix at the 30 °C for 30 min. Subsequently, the end-repaired cDNA was added single A base followed by adapter ligation. We performed PCR with PCR Primer Cocktail and PCR Master Mix to amplify the cDNA. The final library was quantified determining the average molecule length measured by the Agilent 2100 bioanalyzer (Agilent DNA 1000 Reagents) and via QPCR (TaqMan Probe), the cDNA library was eventually sequenced on the HiSeq 2000 System (TruSeq SBS KIT-HS V3,Illumina) in the Beijing Genomics Institute (Shenzhen, China; http://www.genomics.cn/index). Above all, the RNA extraction, library preparation and RNA-Seq were carried out according to descriptions showed by Wang et al. [5] and Wang et al. [111].

### *De novo* assembly and data filtering

Raw reads produced directly from the sequencing platform include dirty reads containing unknown or low-quality bases and adapters. After removing the reads with adaptors, the reads with more than 5 % unknown nucleotides and the low-quality reads (>20 % read rate with a quality value ≤ 10), the remaining clean reads were used for the subsequent analysis [112]. Trinity [112] software was then used to assemble short strips. The sequences resulting from Trinity are called unigenes. Using TGICL [113] to further account for splicing and redundancy, these sequences were assembled into homologous transcript clusters. The same process was applied to all three samples to obtain the longest possible non-redundant unigenes. Based on the clustering of the homologous transcripts, the unigenes were divided into clusters composed of several high-similarity (>70 %) unigenes (starting with CL) and singletons (starting with unigenes). Finally, the unigenes were aligned by BLASTX (E-value < 0.00001) to protein databases (in order of priority) NR, Swiss-Prot, KEGG and COG. The proteins showed the highest ranks in the results of BLAST search were used to determine the coding region sequences of the unigenes. We used ESTS software [114] to determine the sequence direction, if a unigene could not align with any of the above databases. Thereby the nucleotide sequence in the direction from 5’ to 3’ and the amino sequence of the predicted coding region are produced. We have deposited all sequencing data at the sequence read archive of NCBI (Accession number SRR3270364, SRR3270376, SRR3270829, SRR3270928, SRR3270974, SRR3270993, SRR3270997, SRR3271001 and SRR3271002).

### DEGs selection

We used Tags per Million (TPM) to normalize clean tags of each library to obtain the normalized gene expression levels. DEGs were determined using the NOISeq-bio method as previously reported by Tarazona et al. [115]. We estimated the fold changes (log_2_Ratio) on the basis of the normalized gene expression levels in each sample. We used the absolute values of log_2_Ratio > 1 and probability > 0.7 as the thresholds criteria for significant differences in gene expression [31]. In addition, DEGs related to plant hormone metabolism and signaling, TFs and the vesicle polarized trafficking, cell wall biosynthesis, the ubiquitination machinery of the ubiquitin system and species-specific secondary metabolites pathways were mainly analyzed.

### Unigene annotation and classification

Bioinformatics procedures were employed to classify the annotation of unigenes. BLAST was used against the NT, NR, KEGG, Swiss-Prot and COG (E-value ≤ 1.0E-5) databases for annotation, and subsequently the number of unigenes annotated within each database was counted. Based on the NR annotation, GO functional annotations can be obtained. First, the Blast2GO [32] to get the GO annotation of the unigenes, while the WEGO [33] was used to analyze GO functional classifications for all unigenes and determine the distribution of species gene functions at the macro level. The KEGG annotation was employed to get pathway annotations for our unigenes.

### Clustering of gene expression profiles

We performed a hierarchical cluster analysis using the log_2_Ratio of the 125 co-expressed DEGs in the CK-VS-LT and CK-VS-NO expression libraries by cluster [116] and Java Treeview [117] software.

### qRT-PCR analysis

The qRT-PCR analysis was carried out according to the description shown by Wang et al. [111]. The primers used in this assay were shown in Additional file 16: Table S14, and the *C. sinensis* 18S rRNA was employed as the reference.

### Statistical analyses

Statistical analyses were performed as previous reported by Wang et al. [111]. Briefly, all experiments were replicated at least three times, and all data were represented as the means ± standard deviations (SD). Group differences were tested using one-way ANOVA and Duncan’s test, and significant differences are represented by different letters (P < 0.05). Data analysis was performed using SPSS 20 software.

## Additional files

Additional file 1: Figure S1. Assessment of assembly quality. Distribution of mapped reads within the assembled unigenes determined unigene assembly quality. (TIF 2384 kb)
Additional file 2: Table S1. DEGs identified from the comparison between control (CsPT-CK) and 4 °C-treated (CsPT-LT) pollen tbues. All of the samples were replicated three times. CK and LT FPKM: fragments per kb per million reads for each unigene in the CK and LT libraries, respectively. The log_2_Ratio (LT/CK): ratio between the FPKM of LT and CK. The absolute values of log_2_Ratio > 1 and probability > 0.7 were used as threshold for assigning significance. Annotation of DEGs against NR, NT, Swiss-Prot protein, KEGG, COG and GO were all reported in the tables. “-”: no hit. (XLS 381 kb)
Additional file 3: Table S2. DEGs identified from the comparison between control (CsPT-CK) and NO-treated (CsPT-NO) pollen tubes. All of the samples were replicated three times. CK and NO FPKM: fragments per kb per million reads for each unigene in the CK and NO libraries, respectively. The log_2_Ratio (NO/CK): ratio between the FPKM of NO and CK. The absolute values of log_2_Ratio > 1 and probability > 0.7 were used as threshold for assigning significance. Annotation of DEGs against NR, NT, Swiss-Prot protein, KEGG, COG and GO were all reported in the tables. “-”: no hit. (XLS 250 kb)
Additional file 4: Table S3. DEGs identified from the comparison between 4 °C- (CsPT-LT) and NO-treated (CsPT-NO) pollen tubes. All of the samples were replicated three times. LT and NO FPKM: fragments per kb per million reads for each unigene in the LT and NO libraries, respectively. The log_2_Ratio (LT/NO): ratio between the FPKM of LT and CK. The absolute values of log_2_Ratio > 1 and probability > 0.7 were used as threshold for assigning significance. Annotation of DEGs against NR, NT, Swiss-Prot protein, KEGG, COG and GO were all reported in the tables. “-”: no hit. (XLS 451 kb)
Additional file 5: Table S4. GO classification of DEGs identified from the comparison between control (CK) and 4 °C-treated (LT) pollen tubes. (XLS 52 kb)
Additional file 6: Table S5. GO classification of DEGs identified from the comparison between control (CK) and NO-treated (NO) pollen tubes. (XLS 40 kb)
Additional file 7: Table S6. GO classification of DEGs identified from the comparison between pollen tubes exposed to 4 °C (LT) and pollen tubes treated with NO donor (NO). (XLS 59 kb)
Additional file 8: Table S7. The 125 DEGs identified from the comparison between CK-VS-LT and CK-VS-NO. (XLS 89 kb)
Additional file 9: Table S8. DEGs involved in vesicle polarized trafficking and cell wall biosynthesis between CK and LT (CK-VS-LT). The absolute values of log_2_Ratio (LT/CK) > 1 and probability > 0.7 were used as threshold for assigning significance. CK: control; LT: 4 °C treatment. (DOC 37 kb)
Additional file 10: Table S9. DEGs involved in vesicle polarized trafficking and cell wall biosynthesis between CK and NO (CK-VS-NO). The absolute values of log_2_Ratio (NO/CK) > 1 and probability > 0.7 were used as threshold for assigning significance. CK: control; NO: NO treatment. (DOC 31 kb)
Additional file 11: Table S10. DEGs involved in the ubiquitination machinery of the ubiquitin system between CK and LT (CK-VS-LT). The absolute values of log_2_Ratio (LT/CK) > 1 and probability > 0.7 were used as threshold for assigning significance. CK: control; LT: 4 °C treatment. (DOC 55 kb)
Additional file 12: Table S11. DEGs involved in the ubiquitination machinery of the ubiquitin system between CK and NO (CK-VS-NO). The absolute values of log_2_Ratio (NO/CK) > 1 and probability > 0.7 were used as threshold for assigning significance. CK: control; NO: NO treatment. (DOC 37 kb)
Additional file 13: Table S12. DEGs involved in flavonoids, caffeine and theanine biosynthesis pathways between CK and LT (CK-VS-LT). The absolute values of log_2_Ratio (LT/CK) > 1 and probability > 0.7 were used as threshold for assigning significance. CK: control; LT: 4 °C treatment. (DOC 36 kb)
Additional file 14: Table S13. DEGs involved in flavonoids, caffeine and theanine biosynthesis pathways between CK and NO (CK-VS-NO). The absolute values of log_2_Ratio (NO/CK) > 1 and probability > 0.7 were used as threshold for assigning significance. CK: control; NO: NO treatment. (DOC 34 kb)
Additional file 15: Figure S2. GO functional classifications in transcriptome data of *C. sinensis* leaves and pollen tubes under low temperature stress. A: GO functional classifications in transcriptome data of *C. sinensis* leaves under cold stress by Wang et al. [5]. B: GO functional classifications in transcriptome data of *C. sinensis* pollen tubes under cold stress. (TIF 3893 kb)
Additional file 16: Table S14. Primers used in the quantitative real-time PCR (qRT-PCR) analysis of DEGs. (DOC 33 kb)

## Abbreviations

ABA: Abscisic acid
ABA8ox: ABA 8’-hydroxylase
AP2/ERF: APETALA2/ethylene responsive factor
BAK1: BRI1-associated kinase 1
bHLH: Basic helix-loop-helix
BRI1: Brassinosteroid-insensitive 1
BRs: Brassinosteroids
BTB/POZ: Broad complex tramtrack, bric-a-brac/pox virus and zinc finger
CaM: Calmodulin
CAMTA: CaM-binding transcription activator
CBF: C repeat binding factor
cGMP: Guanosine 3’ 5’-cyclic monophosphate
COG: Cluster of Orthologous Groups of proteins database
cPTIO: 2-(4-carboxyphenyl)-4,4,5,5-tetramethylimidazoline-1-oxyl-3-oxide
CRLs: Cullin–RING ligases
CTR1: Constitutive Triple Response 1
DEGs: Differentially expressed genes
E1s: Ubiquitin activating enzymes
E2s: Ubiquitin conjugating enzymes
E3s: Ubiquitin ligases
EBF: EIN3-Binding F-box
EBR: Epibrassinolide
EIL1: EIN3-like1
EIN: Ethylene insensitive
ERS1: Ethylene response sensor 1
ET: Ethylene
GA20x: GA 20oxidase
GA2ox: GA 2-oxidase
GA3x: GA 3oxidase
GAs: Gibberellins
GID1: GA insensitive dwarf 1
GO: Gene Ontology database
HECT: Homology to E6-Associated Carboxyl-Terminus
IAA: Indole-3-acetic acid
KEGG: Kyoto Encyclopedia of Genes and Genomesdatabase
LRR: Leucine-rich repeat
MYB: MYB domain-containing protein
NO: Nitric oxide
NOS: NO synthase
NR: NCBI non-redundant database
NT: Mysql-nt database
PHYA: Phytochrome A
PHYB: Phytochrome B
PI4K: Phosphatidylinositol-4-kinase
PI4P: Phosphatidylinositol-4-phosphate
PIN1: PIN-FORMED 1
PIP2: PI 4,5-bisphosphate
PIP5K: PI 4-phosphate 5-kinase
PLD: Phospholipase D
PP2C: Type 2C protein phosphatase
qRT-PCR: Real-time quantitative polymerase chain reaction
RING: Really Interesting New Gene
RLK: LRR receptor-like kinase
SAMS: S-adenosylmethionine synthetase
SNF1: S-nitrosylation of sucrose non-fermenting 1
SNP: Sodium nitroprusside
SnRK: SNF1-related protein kinase
TAIR: The Arabidopsis Information Resource
TFs: Transcription factors
TPL: TOPLESS
TPM: Tags per Million
UPS: Ubiquitin–26S proteasome system
VAMP726: Vesicle-associated membrane protein 726
WEGO: Web Gene Ontology Annotation Plot
ZFP: Zinc finger protein
