# Supplementary figures and images for: Gene expression profile indicates involvement of NO in Camellia sinensis pollen tube growth at low temperature
Source: BMC Genomics. 2016 Oct 18;17:809. doi: 10.1186/s12864-016-3158-4 (PMC5070194; doi:10.1186/s12864-016-3158-4)

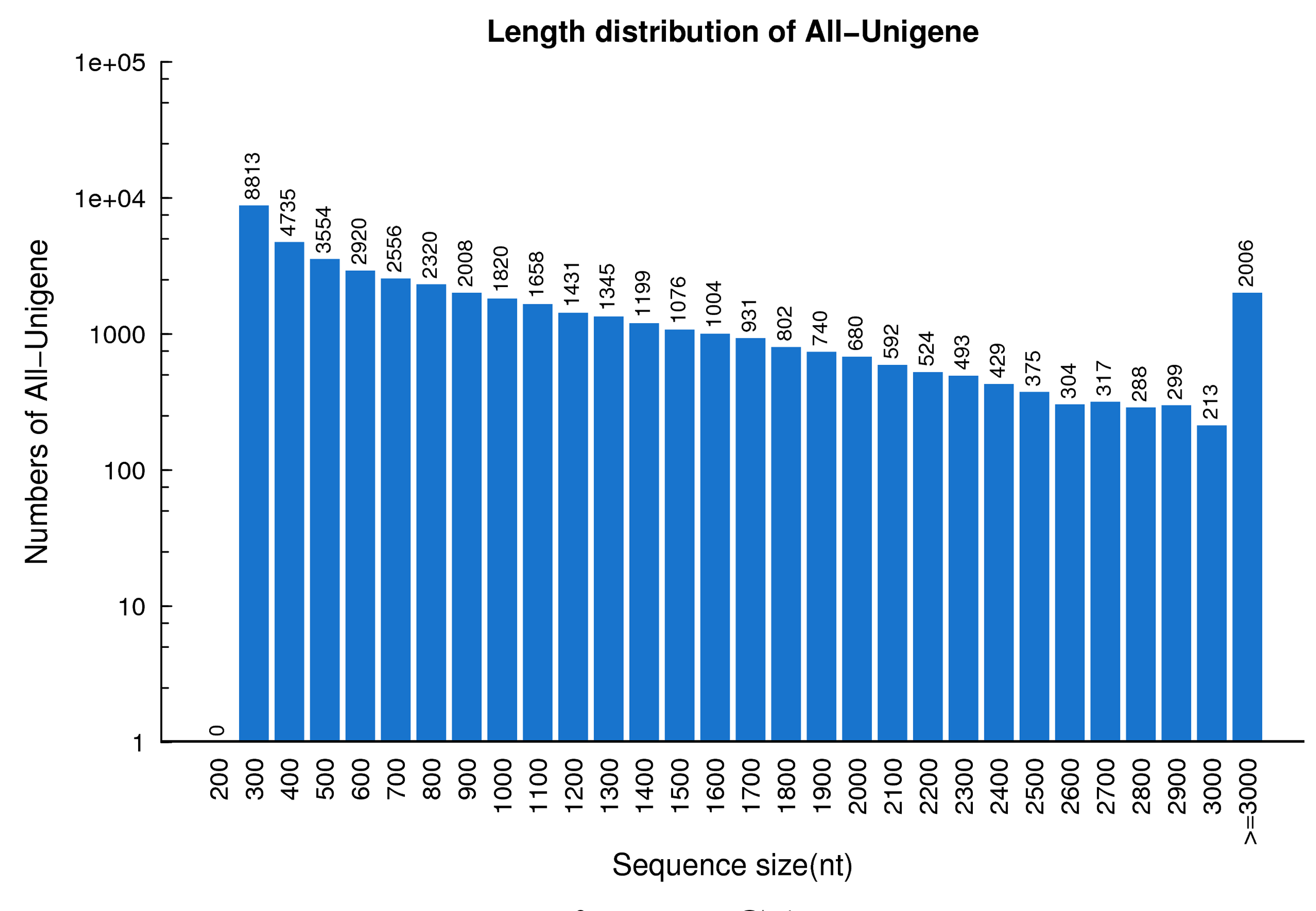

Supplement: Additional file 1: Figure S1. — Assessment of assembly quality. Distribution of mapped reads within the assembled unigenes determined unigene assembly quality. (TIF 2384 kb) [file 12864_2016_3158_MOESM1_ESM.tif]

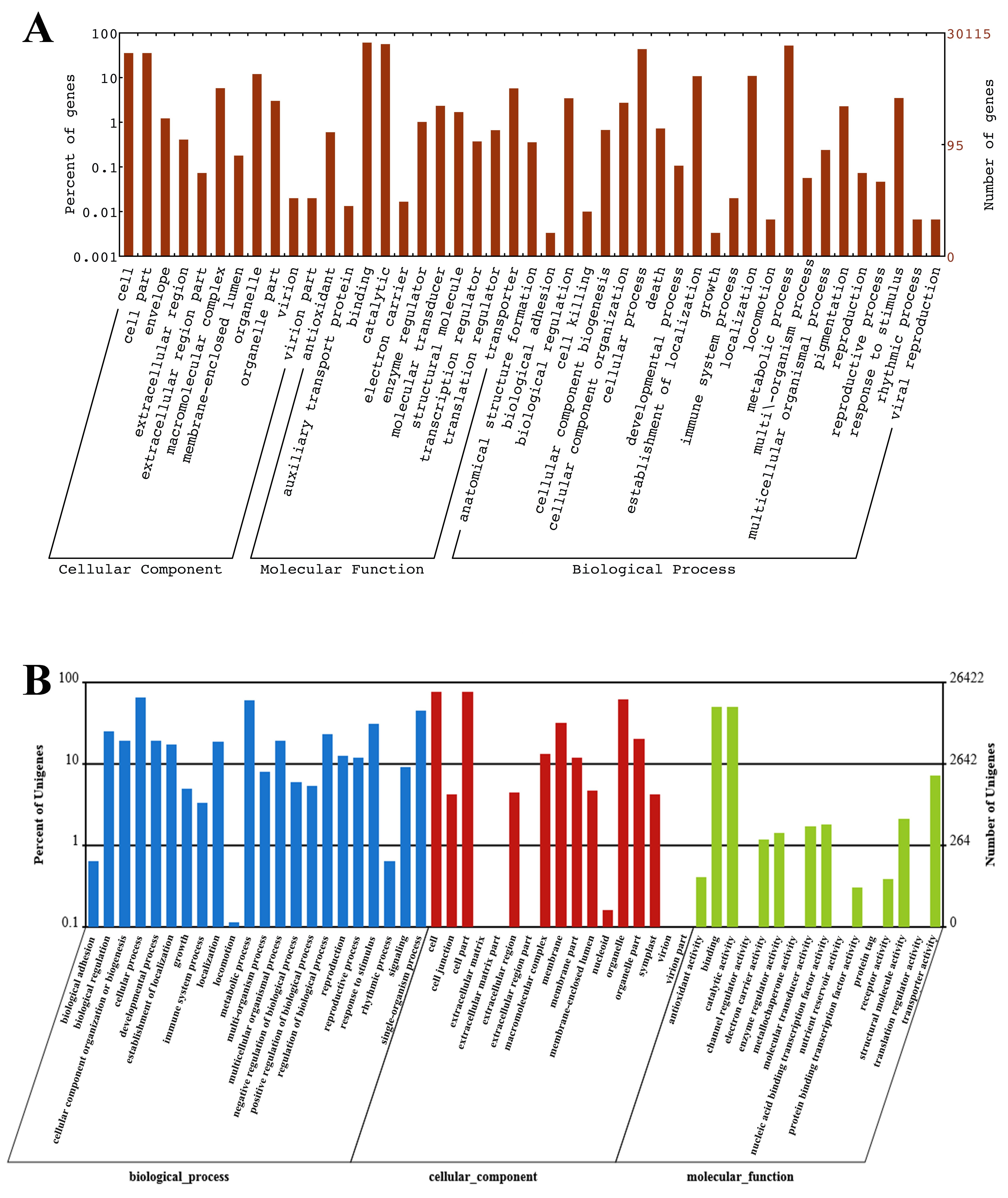

Supplement: Additional file 15: Figure S2. — GO functional classifications in transcriptome data of C. sinensis leaves and pollen tubes under low temperature stress. A: GO functional classifications in transcriptome data of C. sinensis leaves under cold stress by Wang et al. [5]. B: GO functional classifications in transcriptome data of C. sinensis pollen tubes under cold stress. (TIF 3893 kb) [file 12864_2016_3158_MOESM15_ESM.tif]
