# Supplementary material for: Gene expression profile indicates involvement of NO in Camellia sinensis pollen tube growth at low temperature
Source: BMC Genomics. 2016 Oct 18;17:809. doi: 10.1186/s12864-016-3158-4 (PMC5070194; doi:10.1186/s12864-016-3158-4)
Supplement: Additional file 9: Table S8. — DEGs involved in vesicle polarized trafficking and cell wall biosynthesis between CK and LT (CK-VS-LT). The absolute values of log2Ratio (LT/CK) > 1 and probability > 0.7 were used as threshold for assigning significance. CK: control; LT: 4 °C treatment. (DOC 37 kb) [file 12864_2016_3158_MOESM9_ESM.doc]

**Additional file 9: Table S8. DEGs involved in vesicle polarized trafficking and cell wall biosynthesis between CK and LT (CK-VS-LT).**

| GeneID | Gene length | log_2_Ratio(LT/CK) | Up-Down-  Regulation(LT/CK) | Probability | Gene annotation |
| --- | --- | --- | --- | --- | --- |
| CL4306.Contig4_All | 2554 | 1.406501452 | up | 0.785781717 | phosphatidylinositol 4-kinase |
| CL4306.Contig1_All | 2597 | 1.279406067 | up | 0.765460583 | phosphatidylinositol 4-kinase |
| CL4306.Contig3_All | 2616 | 1.101899014 | up | 0.72588849 | phosphatidylinositol 4-kinase |
| Unigene13861_All | 387 | 1.826741741 | up | 0.776363355 | phosphatidylinositol 4-kinase |
| Unigene17835_All | 290 | -1.910036171 | down | 0.759133117 | Phosphatidylinositol  4-phosphate 5-kinase |
| CL2130.Contig1_All | 404 | -1.872199342 | down | 0.707536805 | Vesicle-associated membrane protein |
| CL3822.Contig1_All | 309 | 1.317143797 | up | 0.748327189 | Vesicle-associated membrane protein |
| Unigene10170_All | 210 | -1.265209061 | down | 0.700760924 | COBRA-like protein |

The absolute values of log_2_Ratio (LT/CK) > 1 and probability > 0.7 were used as threshold for assigning significance. CK: control; LT: 4 °C treatment.
