# Supplementary material for: Gene expression profile indicates involvement of NO in Camellia sinensis pollen tube growth at low temperature
Source: BMC Genomics. 2016 Oct 18;17:809. doi: 10.1186/s12864-016-3158-4 (PMC5070194; doi:10.1186/s12864-016-3158-4)
Supplement: Additional file 10: Table S9. — DEGs involved in vesicle polarized trafficking and cell wall biosynthesis between CK and NO (CK-VS-NO). The absolute values of log2Ratio (NO/CK) > 1 and probability > 0.7 were used as threshold for assigning significance. CK: control; NO: NO treatment. (DOC 31 kb) [file 12864_2016_3158_MOESM10_ESM.doc]

**Additional file 10: Table S9 DEGs involved in vesicle polarized trafficking and cell wall biosynthesis between CK and NO (CK-VS-NO)**

| GeneID | Gene length | log_2_Ratio(NO/CK) | Up-Down-  Regulation(NO/CK) | Probability | Gene annotation |
| --- | --- | --- | --- | --- | --- |
| Unigene13861_All | 387 | 1.863723506 | up | 0.700149864 | phosphatidylinositol 4-kinase |
| CL1623.Contig1_All | 481 | -1.144430031 | down | 0.750318461 | Vesicle-associated membrane protein |
| Unigene10170_All | 210 | -2.501471401 | down | 0.759780254 | COBRA-like protein |

The absolute values of log_2_Ratio (NO/CK) > 1 and probability > 0.7 were used as threshold for assigning significance. CK: control; NO: NO treatment.
