# Supplementary material for: Gene expression profile indicates involvement of NO in Camellia sinensis pollen tube growth at low temperature
Source: BMC Genomics. 2016 Oct 18;17:809. doi: 10.1186/s12864-016-3158-4 (PMC5070194; doi:10.1186/s12864-016-3158-4)
Supplement: Additional file 11: Table S10. — DEGs involved in the ubiquitination machinery of the ubiquitin system between CK and LT (CK-VS-LT). The absolute values of log2Ratio (LT/CK) > 1 and probability > 0.7 were used as threshold for assigning significance. CK: control; LT: 4 °C treatment. (DOC 55 kb) [file 12864_2016_3158_MOESM11_ESM.doc]

**Additional file 11: Table S10. DEGs involved in the ubiquitination machinery of the ubiquitin system between CK and LT (CK-VS-LT)**

| GeneID | Gene length | log_2_Ratio(LT/CK) | Up-Down-  Regulation(LT/CK) | Probability | Gene annotation |
| --- | --- | --- | --- | --- | --- |
| Unigene979_All | 580 | -1.671614153 | down | 0.791531369 | E2 |
| Unigene6536_All | 276 | -2.74006362 | down | 0.742543356 | E3 |
| Unigene9475_All | 382 | 1.147076601 | up | 0.709884681 | E3 |
| Unigene1207_All | 496 | -1.743612399 | down | 0.70614517 | E3 |
| Unigene1806_All | 288 | -1.638495223 | down | 0.791842249 | E3 |
| Unigene21945_All | 297 | -1.680904825 | down | 0.748887098 | 26S proteasome |
| Unigene11403_All | 1295 | -2.563240978 | down | 0.826220529 | U-box |
| CL2938.Contig2_All | 1422 | -3.475857582 | down | 0.912872222 | U-box |
| Unigene19641_All | 212 | 1.549227277 | up | 0.703931574 | U-box |
| Unigene4730_All | 365 | -2.208240249 | down | 0.713270181 | U-box |
| Unigene19089_All | 1563 | -4.409442838 | down | 0.945827942 | F-box |
| CL5173.Contig1_All | 567 | -2.576699132 | down | 0.807116059 | F-box |
| CL2610.Contig1_All | 1080 | -1.518999731 | down | 0.801879115 | F-box |
| CL245.Contig2_All | 1576 | -1.738196534 | down | 0.783458255 | F-box |
| Unigene6174_All | 400 | -2.391789851 | down | 0.721139839 | F-box |
| Unigene14012_All | 211 | -5.788995121 | down | 0.713599779 | F-box |
| Unigene17670_All | 214 | -1.91872275 | down | 0.70682878 | F-box |
| Unigene15602_All | 216 | 1.668436013 | up | 0.70504651 | F-box |
| Unigene1330_All | 355 | 1.618869102 | up | 0.704729119 | F-box |
| Unigene17396_All | 292 | 2.283987044 | up | 0.813256346 | BTB/POZ domain-containing protein |
| Unigene14777_All | 1035 | -1.389083417 | down | 0.77360694 | BTB/POZ domain-containing protein |
| Unigene11272_All | 680 | -1.307248159 | down | 0.756668864 | BTB/POZ domain-containing protein |

The absolute values of log_2_Ratio (LT/CK) > 1 and probability > 0.7 were used as threshold for assigning significance. CK: control; LT: 4 °C treatment.
