# Supplementary material for: Gene expression profile indicates involvement of NO in Camellia sinensis pollen tube growth at low temperature
Source: BMC Genomics. 2016 Oct 18;17:809. doi: 10.1186/s12864-016-3158-4 (PMC5070194; doi:10.1186/s12864-016-3158-4)
Supplement: Additional file 12: Table S11. — DEGs involved in the ubiquitination machinery of the ubiquitin system between CK and NO (CK-VS-NO). The absolute values of log2Ratio (NO/CK) > 1 and probability > 0.7 were used as threshold for assigning significance. CK: control; NO: NO treatment. (DOC 37 kb) [file 12864_2016_3158_MOESM12_ESM.doc]

**Additional file 12: Table S11. DEGs involved in the ubiquitination machinery of the ubiquitin system between CK and NO (CK-VS-NO)**

| GeneID | Gene length | log_2_Ratio(NO/CK) | Up-Down-  Regulation(NO/CK) | Probability | Gene annotation |
| --- | --- | --- | --- | --- | --- |
| Unigene6321_All | 304 | 2.86196367 | up | 0.813920246 | E3 |
| Unigene13761_All | 593 | 1.921009849 | up | 0.773440682 | E3 |
| Unigene6536_All | 276 | -2.025411827 | down | 0.796765707 | E3 |
| Unigene21311_All | 254 | 1.832890399 | up | 0.753288863 | E3 |
| Unigene21945_All | 297 | -1.632792442 | down | 0.753587776 | 26S proteasome |
| Unigene11403_All | 1295 | -2.563240978 | down | 0.826220529 | U-box |
| Unigene11905_All | 458 | 1.817567998 | up | 0.756035283 | U-box |
| Unigene21358_All | 205 | 2.22806402 | up | 0.756939354 | F-box |
| Unigene17396_All | 292 | 1.580768457 | up | 0.757528222 | BTB/POZ domain-containing protein |

The absolute values of log_2_Ratio (NO/CK) > 1 and probability > 0.7 were used as threshold for assigning significance. CK: control; NO: NO treatment.
