# Supplementary material for: Gene expression profile indicates involvement of NO in Camellia sinensis pollen tube growth at low temperature
Source: BMC Genomics. 2016 Oct 18;17:809. doi: 10.1186/s12864-016-3158-4 (PMC5070194; doi:10.1186/s12864-016-3158-4)
Supplement: Additional file 13: Table S12. — DEGs involved in flavonoids, caffeine and theanine biosynthesis pathways between CK and LT (CK-VS-LT). The absolute values of log2Ratio (LT/CK) > 1 and probability > 0.7 were used as threshold for assigning significance. CK: control; LT: 4 °C treatment. (DOC 36 kb) [file 12864_2016_3158_MOESM13_ESM.doc]

**Additional file 13: Table S12. DEGs involved in flavonoids, caffeine and theanine biosynthesis pathways between CK and LT (CK-VS-LT)**

| GeneID | Gene length | log_2_Ratio(LT/CK) | Up-Down-  Regulation(LT/CK) | Probability | Gene annotation |
| --- | --- | --- | --- | --- | --- |
| Unigene10059_All | 410 | -2.225010376 | down | 0.720439952 | Phenylalanine ammonia-lyase |
| CL1933.Contig2_All | 218 | -3.617850406 | down | 0.736606525 | Isoflavone 2'-hydroxylase |
| Unigene4423_All | 214 | -2.209226087 | down | 0.773586595 | AMP deaminase |
| Unigene1362_All | 303 | -1.324109419 | down | 0.72780911 | caffeine synthase |
| Unigene13038_All | 380 | -1.279190272 | down | 0.748683643 | glutamine synthetase |
| CL4694.Contig1_All | 268 | -2.118342447 | down | 0.777590599 | Glutamate receptor |
| Unigene21753_All | 206 | -2.980851843 | down | 0.759002905 | Pyruvate kinase |

The absolute values of log_2_Ratio (LT/CK) > 1 and probability > 0.7 were used as threshold for assigning significance. CK: control; LT: 4 °C treatment.
