# Supplementary material for: Gene expression profile indicates involvement of NO in Camellia sinensis pollen tube growth at low temperature
Source: BMC Genomics. 2016 Oct 18;17:809. doi: 10.1186/s12864-016-3158-4 (PMC5070194; doi:10.1186/s12864-016-3158-4)
Supplement: Additional file 14: Table S13. — DEGs involved in flavonoids, caffeine and theanine biosynthesis pathways between CK and NO (CK-VS-NO). The absolute values of log2Ratio (NO/CK) > 1 and probability > 0.7 were used as threshold for assigning significance. CK: control; NO: NO treatment. (DOC 34 kb) [file 12864_2016_3158_MOESM14_ESM.doc]

**Additional file 14: Table S13. DEGs involved in flavonoids, caffeine and theanine biosynthesis pathways between CK and NO (CK-VS-NO)**

| GeneID | Gene length | log_2_Ratio(NO/CK) | Up-Down-  Regulation(NO/CK) | Probability | Gene annotation |
| --- | --- | --- | --- | --- | --- |
| Unigene4760_All | 360 | -2.637254211 | down | 0.81854241 | caffeine synthase 1 |
| CL1512.Contig4_All | 432 | 1.280919538 | up | 0.750798189 | caffeine synthase 1 |
| Unigene1362_All | 303 | -1.347491297 | down | 0.745846161 | caffeine synthase 1 |
| CL4694.Contig1_All | 268 | -2.855122548 | down | 0.838525632 | glutamate receptor |
| CL5762.Contig1_All | 290 | -2.302395184 | down | 0.707239896 | pyruvate kinase |

The absolute values of log_2_Ratio (NO/CK) > 1 and probability > 0.7 were used as threshold for assigning significance. CK: control; NO: NO treatment.
