# Supplementary material for: Gene expression profile indicates involvement of NO in Camellia sinensis pollen tube growth at low temperature
Source: BMC Genomics. 2016 Oct 18;17:809. doi: 10.1186/s12864-016-3158-4 (PMC5070194; doi:10.1186/s12864-016-3158-4)
Supplement: Additional file 16: Table S14. — Primers used in the quantitative real-time PCR (qRT-PCR) analysis of DEGs. (DOC 33 kb) [file 12864_2016_3158_MOESM16_ESM.doc]

**Additional file 15: Table S14. Primers used in the quantitative real-time PCR (qRT-PCR) analysis of DEGs**

| GeneID | Forward primer | Reverse primer | Gene annotation |
| --- | --- | --- | --- |
| Unigene2215_All | CTGCGAAGTAAAGGTTAGAAA | GGAAAGTTAGAAAGCGATGTT | CAMTA |
| Unigene21920_All | GTTGGCAATCAATCCCTCCG | GTGTGAGCGGCATTACCTGAA | zinc finger protein |
| CL2776.Contig1_All | GCTAAATAGGCTTGTTGTCCCA | CACCTCATCTACCTTCCCACC | AP2/ERF |
| CL2425.Contig1_All | CTCGACGCACCAGCTAACAC | CACCCAAACCAAATGGAACA | Cytochrome P450 |
| Unigene8699_All | CCTACTTGGCATTCCATCCG | AAGCCCTGTGATTCGTTTCTG | TOPLESS |
| Unigene22243_All | TCCAAGATGTCCCTCTGCTA | ATCACGGAAATCCCAAGTC | Phospholipase D |
| CL10.Contig2_All | GATATTAAGCTCATTACATCATCC | TCCGTATAAACTGTTTCAAAGATC | Serine/threonine-protein kinase |
| Unigene17702_All | CAAGATTCCAAACTTGAATTTTCA | AATACTTTTCAATTTCCTCTGCAG | MYB |
| CL580.Contig48_ | AAGATCTTAAACGAGTTTCTATGG | AAGCAGTTCACTGCAACTATATAC | PP2C |
